# FMNH_2_-dependent monooxygenases initiate catabolism of sulfonamides in *Microbacterium* sp. strain BR1 subsisting on sulfonamide antibiotics

**DOI:** 10.1038/s41598-017-16132-8

**Published:** 2017-11-17

**Authors:** Benjamin Ricken, Boris A. Kolvenbach, Christian Bergesch, Dirk Benndorf, Kevin Kroll, Hynek Strnad, Čestmír Vlček, Ricardo Adaixo, Frederik Hammes, Patrick Shahgaldian, Andreas Schäffer, Hans-Peter E. Kohler, Philippe F.-X. Corvini

**Affiliations:** 1 Institute for Ecopreneurship, School of Life Sciences, University of Applied Sciences and Arts Northwestern Switzerland, Muttenz, Switzerland; 2 Bioprocess Engineering, Max Planck Institute for Dynamics of Complex Technical Systems, Magdeburg, Germany; 3 Laboratory of Genomics and Bioinformatics, Institute of Molecular Genetics of the Academy of Sciences of the Czech Republic, Prague, Czech Republic; 4 Department Environmental Microbiology, Swiss Federal Institute of Aquatic Science and Technology, Dübendorf, Switzerland; 5 Institute for Chemistry and Bioanalytics, School of Life Sciences, University of Applied Sciences and Arts Northwestern Switzerland, Muttenz, Switzerland; 6 Institute for Environmental Research, RWTH Aachen University, Aachen, Germany

## Abstract

We report a cluster of genes encoding two monooxygenases (SadA and SadB) and one FMN reductase (SadC) that enable *Microbacterium* sp. strain BR1 and other Actinomycetes to inactivate sulfonamide antibiotics. Our results show that SadA and SadC are responsible for the initial attack of sulfonamide molecules resulting in the release of 4-aminophenol. The latter is further transformed into 1,2,4-trihydroxybenzene by SadB and SadC prior to mineralization and concomitant production of biomass. As the degradation products lack antibiotic activity, the presence of SadA will result in an alleviated bacteriostatic effect of sulfonamides. In addition to the relief from antibiotic stress this bacterium gains access to an additional carbon source when this gene cluster is expressed. As degradation of sulfonamides was also observed when *Microbacterium* sp. strain BR1 was grown on artificial urine medium, colonization with such strains may impede common sulfonamide treatment during co-infections with pathogens of the urinary tract. This case of biodegradation exemplifies the evolving catabolic capacity of bacteria, given that sulfonamide bacteriostatic are purely of synthetic origin. The wide distribution of this cluster in Actinomycetes and the presence of *traA* encoding a relaxase in its vicinity suggest that this cluster is mobile and that is rather alarming.

## Introduction

Antimicrobial resistance has become a worldwide clinical threat and can lead to a health crisis by 2050^1^. Recent metagenomic analysis of ancient DNA showed that antibiotic resistance is a natural phenomenon that predates the modern selective pressure of clinical antibiotic use^2^. The antibiotic resistome^3^ can be considered a global pool of potential antibiotic resistance genes^4^ and “comprises all of the antibiotic resistance genes including precursor genes that encode proteins with modest antibiotic resistance functionality, or affinity to antibiotics, that might evolve into effective resistance genes”^3,5^.

Contrarily to antibiotics that originate from natural products, the structural moieties of sulfonamides are virtually absent from naturally occurring compounds^6^. Sulfonamides were introduced into the environment around 1935 and microorganisms did not have millennia to evolve resistances. Nevertheless, sulfapyridine resistant bacteria were already reported in 1941^7,8^. Resistance to sulfonamides is mostly related to *sul* genes encoding modified dihydropteroate synthases^9,10^.

There are still large gaps in our understanding of antibiotic resistance evolution and propagation^11^. The relevance of genes encoding enzymes involved in catabolism of antibiotics for antibiotic resistance has been controversially discussed in the literature^12–14^. Although studies indicated that certain microorganisms could use antibiotics as carbon and energy source^15–19^, so far, no gene or enzyme involved in antibiotic subsistence has been identified^12^.

Here, we show that a two-component flavin-dependent monooxygenase (SadA) is initiating catabolism of sulfonamides in *Microbacterium* sp. strain BR1 and portend that genes encoding the respective catabolic enzymes are part of the resistome.

**Figure 1:**
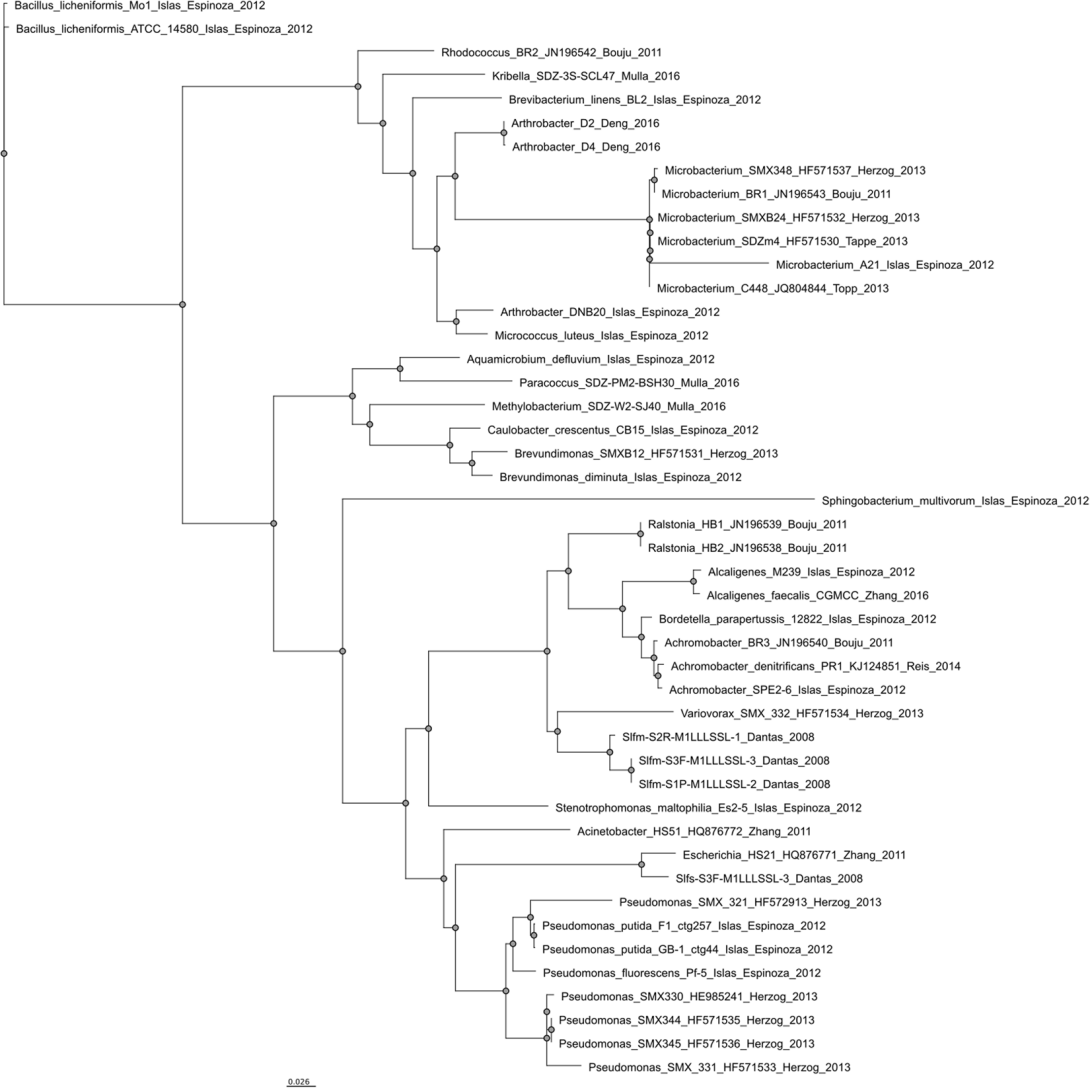
Phylogenetic tree of sulfamethoxazole (SMX) mineralizing and degrading bacterial isolates. All bacteria described in literature with the capability to degrade one or more sulfonamide antibiotics^22–33^ were phylogenetically ordered based on 16S rRNA gene sequences. The 16S rRNA gene sequences of the isolates identified by Islas-Espinoza and colleagues^25^ were not available, and only the sequences of the closest relatives were used (accession numbers of relatives were received by personal communication). Ugene 1.26^54^ was used as the graphical user interface for the bioinformatic tools. Sequences were aligned with MAFFT^61^ (advanced options were inactivated). The tree was built by maximum likelihood with PhyML 3.0^62^ (Substitution model: HKY85, equilibrium frequencies: optimized, number of substitution rate categories: 4, fast likelihood-based method: aLRT, tree improvement: SRT & NNI) and plotted with Ugene. Retrieved scalable vector graphics were annotated with Inkscape 0.91.1.

In a previous study we have shown that *Microbacterium* sp. strain BR1 initiates the degradation of various sulfonamides through a *ipso*-hydroxylation mechanism^20^. To rule out an artefactual growth of bacteria, we used ^14^C-labelled antibiotics to measure ^14^CO_2_ as an unequivocal proof of mineralization, which is expected to reflect their metabolism as officially defined by IUPAC. The fragmentation initiated by *ipso*-hydroxylation destroys the molecular integrity of the parent compounds leading to the simultaneous and irreversible loss of antibiotic activity^21^. In the further course of the metabolic pathway, 4-aminophenol is transformed to 1,2,4-trihydroxybenzene prior to further degradation^21^. Various bacteria capable of metabolizing or degrading sulfonamide antibiotics have been reported^22–33^ (compare Fig. 1). As of now, two studies have brought forth publicly available draft genomes of sulfonamide mineralizing isolates, yet no experimental and true evidence has been presented concerning the enzymes involved in the degradation of sulfonamides as they were merely annotated based on sequence similarities^23,34^.

In the present study, we aimed to identify the genes in *Microbacterium* sp. strain BR1 responsible for catabolism of sulfonamides.

## Results and Discussion

We could verify that carbon of sulfamethoxazole [aniline ring-^14^C(U)] (^14^C-SMX, radiochemical purity: 98.9%, Hartmann Analytic, Germany) was incorporated into the biomass of *Microbacterium sp*. strain BR1. This strain was cultivated in a medium containing SMX devoid of any other sources of carbon. Results showed that under these culture conditions up to 9.33% of the initially applied radioactivity was recovered in the biomass fraction (Supplementary Figure S1). This confirmed an assumption made previously that *Microbacterium* sp. strain BR1 is able to utilize the aniline moiety of sulfonamides for growth^20^.

In order to identify the enzymes responsible for the initial attack on sulfonamides, the genome of *Microbacterium* sp. strain BR1 was sequenced *de novo* by next generation shotgun sequencing (BioProject PRJNA394223). The retrieved draft genome sequence of *Microbacterium* sp. strain BR1 consists of 10 contigs in one scaffold amounting to a total of 3′817′849 bp with a high GC content of 68.1%.

**Figure 2:**
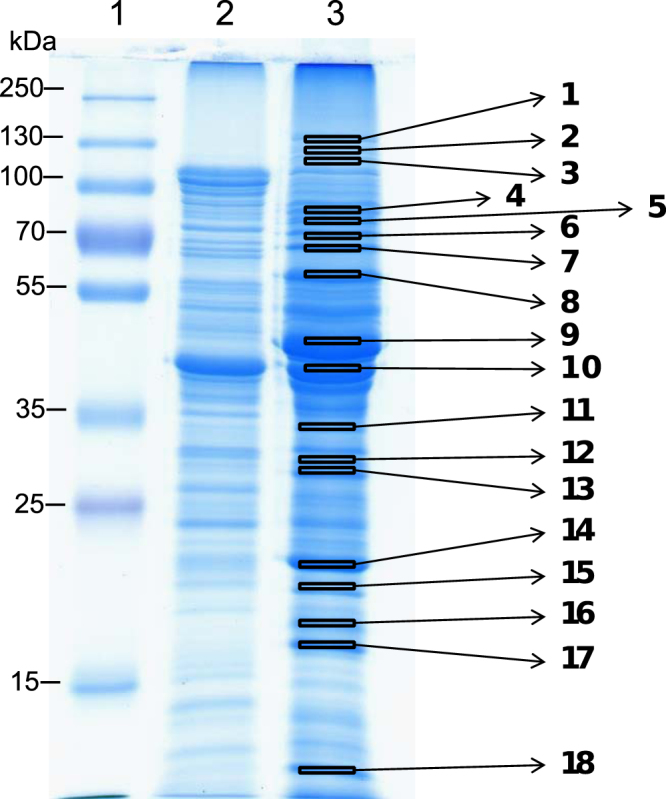
SDS PAGE of *Microbacterium* sp. strain BR1 growing on succinate (lane 2) and SMX (lane 3). 25 µg protein were loaded per lane. The gels were stained with Coomassie Brilliant Blue. Lane 1: molecular weight standard. Excised bands submitted to tryptic digestion and subsequent identification with nano-HPLC are marked with numbers.

Hypothesizing that genes responsible for sulfonamide assimilation are expressed at significantly higher levels when *Microbacterium* sp. strain BR1 is grown in the presence of these antibiotics, we tested whether its cultivation in presence (with 2 mM SMX in MMO medium, described below) or absence (with 2 mM succinate in MMO medium) of sulfonamide leads to remarkable differences in the proteomic pattern of this bacterium. The respective cell lysates of both treatments were subjected to SDS-PAGE. Protein bands that were more intensive in samples containing SMX were cut from the gel and analysed by LC-ESI-ion trap-MS (Fig. 2).

**Figure 3:**
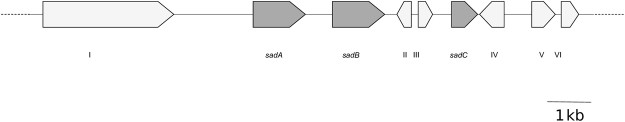
Sad gene cluster responsible for sulfonamide degradation in *Microbacterium* sp. strain BR1. Dark grey genes were identified and cloned in this study to verify their functionality. Light grey genes were annotated with Rapid Annotation of microbial genomes using Subsystems (RAST)^55,56^, but no function has been proven. The genes *sadA, sadB* and *sadC* encode two flavin-dependent monooxygenases and a flavin reductase, respectively. I encodes for a traA like relaxase, putatively involved in gene transfer; II and III are encoding hypothetical proteins; IV encodes a enoyl-CoA dehydrogenase and V and VI are encoding transcriptional regulators belonging to the Cro/CI and yjgF family, respectively).

We matched the resulting MS data to protein sequences generated from genome data obtained for *Microbacterium* sp. strain BR1 (Supplementary Table S3). The retrieved sequences of putative ORFs were matched against protein sequences of *Actinobacteria* (taxid:201174) in the NCBI-nr database by means of the Basic Local Alignment Search Tool (BLAST)^35^. Among the identified ORFs there were two ORFs encoding for putative two-component flavin monooxygenases (designated *sadA* and *sadB*, respectively), which were co-located in a cluster also bearing an ORF (*sadC*) encoding for a flavin reductase (FMNR, designated SadC) (Fig. 3).

The co-localization of a FMN-reductase together with the two flavin dependent monooxygenases were consistent with the results of enzymatic assays carried out using partially purified protein fractions, where addition of NADH as well as FMN and a commercially available FMNR from *E. coli* was essential to the transformation of SMX into 4-aminophenol (Supplementary Figure S4 and Supplementary info chapter “Sequential purification of SadA”). Furthermore, the analysis of a prominent band corresponding to approximately 45 kDa on an SDS-PAGE under denaturing conditions (and 190 kDa according to size exclusion chromatography analysis) of partially purified “sulfonamide monooxygenase” activity indicated that SadA was the enzyme catalysing the initial *ipso*-hydroxylation (Supplementary Figure S5). Both the comparative proteomic analyses and the *de novo* protein sequencing of a partially purified fraction containing sulfonamide monooxygenase activity supported the notion that SadA and SadB were the candidate enzymes involved in the initial reactions of the sulfonamide assimilation by *Microbacterium* sp. strain BR1.

**Figure 4:**
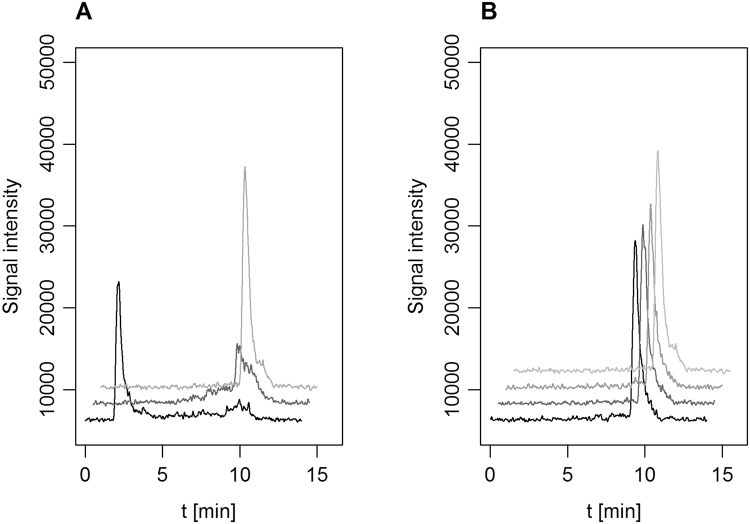
Degradation of ^14^C-SMX by *E. coli* AE expressing the *sad* genes. Peaks eluting around 9.5 minutes correspond to original ^14^C-SMX. (**A**) From black to light grey and front to back, respectively: *E. coli* AE *sadA*; mixture of *E. coli* AE *sadA* and *E. coli* AE *sadB*; buffer control. For incubations of *E. coli* AE *sadA* with ^14^C-SMX, radioactivity co-eluted with the injection peak, which could be attributed to 4AP. (**B**) From black to light grey: not transformed E*. coli* AE; *E. coli* AE *sadC*; *E. coli* AE *sadB*; buffer control.

In order to definitively demonstrate that these enzymes are responsible for catabolism of sulfonamides in *Microbacterium* sp. strain BR1, we heterologously expressed the genes encoding for the flavin-monooxygenases SadA and SadB and the FMNR SadC. Prior to cloning and transformation into *E. coli* Arctic Express (DE3), codon optimization and the addition of an N-terminal HIS-SUMO tag were carried out^36^ to reduce the formation of inclusion bodies. The resulting strains were tested for their capability to degrade ^14^C-SMX. The latter was only degraded by *E. coli* cells expressing *sadA* and a ^14^C-metabolite was formed (Fig. 4), which was identified as 4-aminophenol by HPLC-MS after derivatization (Supplementary Figure S6).

**Figure 5:**
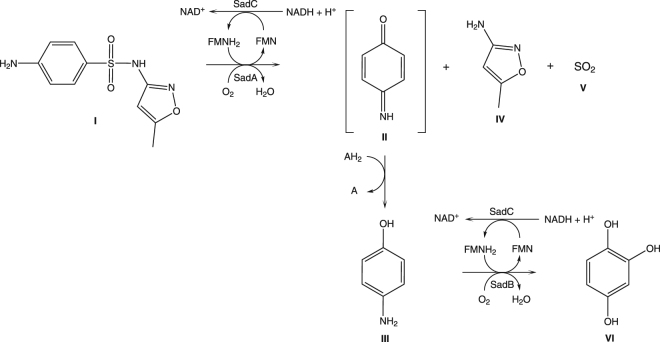
Degradation reactions of SMX catalysed by the *sad* genes. **I**: sulfamethoxazole; **II**, 4-benzoquinone-imine; **III**,-aminophenol; **IV**, 3-amino-5-methylisoxazole; **V**, sulphite; **VI**, trihydroxybenzene.

No changes in SMX concentration were observed when ^14^C-SMX was incubated with *E. coli* expressing either *sadB* or *sadC*. In mixed cultures of *E. coli* expressing *sadA* and *sadB* genes, ^14^C-SMX was degraded and no accumulation of any ^14^C-metabolite was detected. This suggested that the *sadB* gene is responsible for the SMX downstream pathway and the encoded enzyme most likely oxidizes 4-aminophenol to 1,2,4-trihydroxybenzene (neither hydroquinone nor benzoquinone intermediates could be detected) (Supplementary Figure S8 and SI chapter: Degradation studies of 4-aminophenol with *E. coli* *sadB*). It is worth mentioning that both two-component monooxygenases were functionally active in *E. coli* even though the *sadC* gene encoding the FMNR delivering reduced FMN was not cloned along with *sadA* or *sadB*. As FMNH_2_ is essential for both monooxygenases to activate molecular oxygen^37^, an FMNR from *E. coli* must have taken over the role of SadC. The degradation steps catalysed by the sad cluster are illustrated in Fig. 5.

We performed *in silico* analyses to find indications about the relevance of the identified enzymes in other organisms. Both SadA and SadB (which share 38% sequence identity among each other) shared the same closest relative represented in the curated Swiss-Prot database^38^, which is a 3-hydroxy-9,10-secoandrosta-1,3,5(10)-triene-9,17-dione hydroxylase related to cholesterol degradation from the Actinobacterium *Rhodococcus jostii* strain RAH1 (Swiss-Prot: Q0S811) with 30.1 and 31.9% identity, respectively (Supplementary Table S3). The closest relative of the FMNR in the Swiss-Prot database was a flavin reductase found in *Aminobacter aminovorans* with 28% identity. Even though there were only low identities to other enzymes in the Swiss-Prot DB, we found nearly identical gene clusters in the published draft genomes of sulfonamide degraders *Microbacterium* sp. strain C448, *Microbacterium* sp. strain SDZm4 (DSM 18910) and *Arthrobacter* sp. strain D2. *Microbacterium* sp. strain C448 (PRJNA170195) was isolated from an agricultural soil in London (Canada)^34^. *Arthrobacter* sp. strain D2 (PRJNA314012) has been isolated from activated sludge of a municipal wastewater treatment plant in Hong Kong (China)^23^. Additionally, we sequenced the genome of *Microbacterium* sp. strain SDZm4 (DSM 18910), a strain isolated during lysimeter studies in Germany^39^. The *sadB* and *sadC* genes have high identities among all four isolates (Supplementary Figure S2). *sadA* has 99% (*Microbacterium* sp. strain SDZm4) and 97% (*Microbacterium* sp. strain C448) identity compared to that of *Microbacterium* sp. strain BR1. BLAST analysis using *sadA* as query in the draft genome of *Arthrobacter* sp. strain D2 yielded two fragments, one of which aligned to *sadA* with 98% sequence identity, the other, larger one, partially aligned with 82% sequence identity due to significant variation in the 5′end. We assume that the low similarity might result from an assembling artefact rather than an actual sequence variation.

As already mentioned, *Microbacterium* sp. strain BR1, *Microbacterium* sp. strain SDZm4 and *Microbacterium* sp. strain C448 are capable of partially mineralizing sulfonamide antibiotics. Based on our findings we assert that *ipso-*hydroxylation catalysed by SadA and SadA homologues initiate catabolism of sulfonamides in the three *Microbacterium* strains and in *Arthrobacter* sp. strain D2. This molecular mechanism provides an explanation for the growth of these bacteria in presence of sulfonamides and the formation of heterocycle-containing compounds of the parent sulfonamides, which were previously identified as the main metabolites of sulfonamide degradation^20,23,30,39^. This finding implies that the previously unknown genes (identified in the present study) responsible for sulfonamide catabolism are nearly identical in bacterial strains isolated from distant sites on different continents. This suggests that this general sulfonamide degradation mechanism is prevalent and geographically widely spread. Around 30% of the isolates from different soils and activated sludge samples are members of the phylum of Actinobacteria (i.e. the *Rhodococcus*, *Kribella*, *Brevibacterium*, *Arthrobacter*, *Microbacterium* and *Micrococcus* species, respectively, shown in Fig. 1). Compared to the average abundance of Actinobacteria found in soil or sludge consortia, it seems that this phylum is rather overrepresented and especially predisposed to bring forth sulfonamide degraders^40,41^. One reason for this may be the broad metabolic potential of Actinobacteria^42^ in general and of the family of Micrococcaceae in particular^43^.

In the upstream proximity of the SMX-gene cluster, BLAST queries identified sequences providing gene mobility, *e.g*. encoding for a relaxase (Fig. 3). Additionally, the clusters found in all strains were of high identity even in intergenic regions (Supplementary Figure S2). This indicates that the *sad* cluster as a whole might be mobile with a potential to spread as a resistance determinant. The potential of this cluster to act as a resistance determinant should be kept in mind when testing antibiotic mineralizing strains for bioremediating contaminated soils or waters^44,45^. It has recently also been reported by Vila-Costa *et al*. that sulfonamide degradation may act as a resistance mechanism of microbial communities in aquatic mesocosms^46^. So far, we have found the genes (and their nearest relatives in the non-redundant protein database of NCBI) only in genera belonging to the GC rich actinobacteria. Further targeted screening for the genes of the SMX cluster in environmental and clinical samples is necessary to estimate the mobility of the identified genes among different phyla and to assess whether the resistance potential of SadA will be realized in the future.

**Figure 6:**
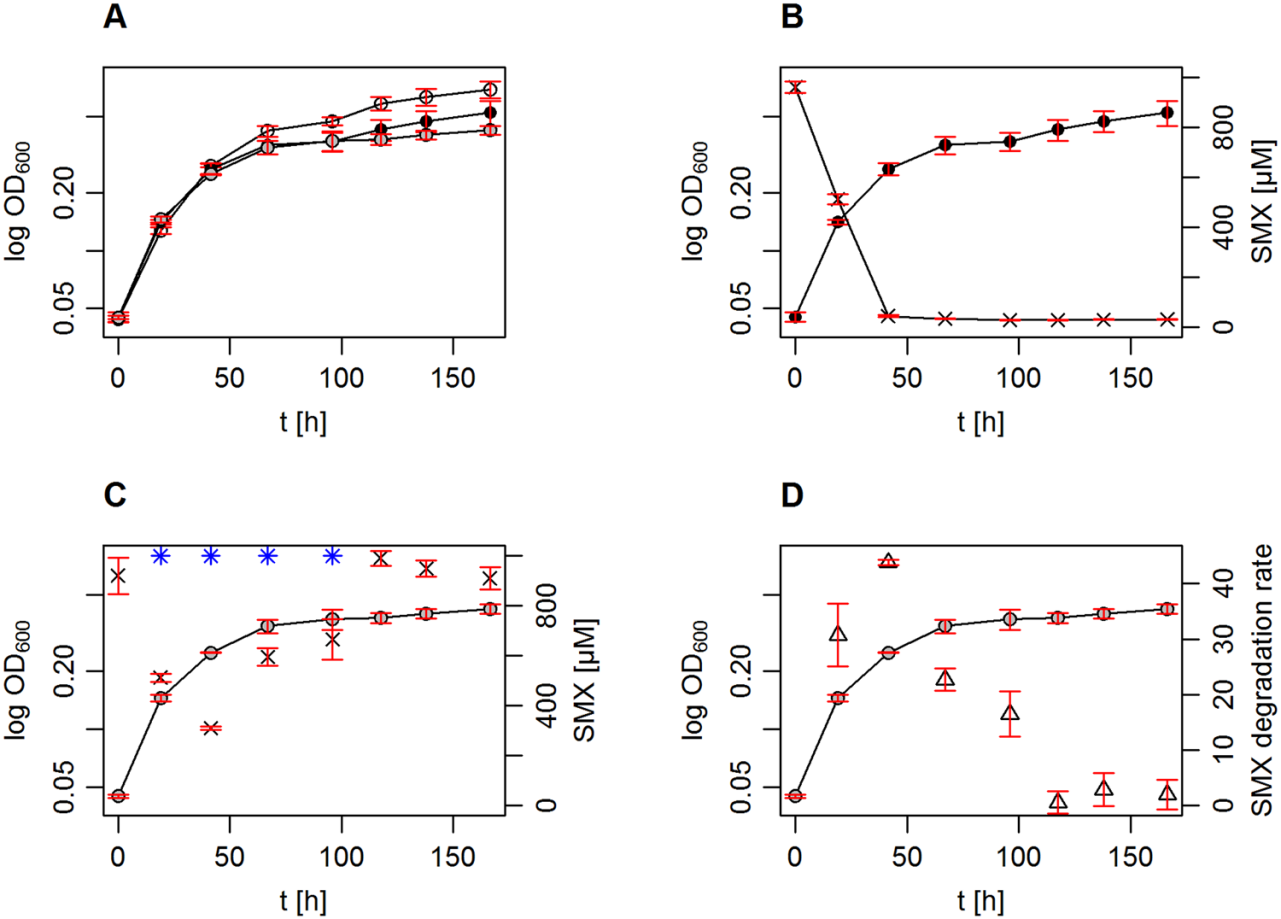
Growth and SMX degradation of *Microbacterium* sp. strain BR1 in artificial urine. The growth of *Microbacterium* sp. strain BR1 was monitored in AUM by OD_600_ measurements. *Microbacterium* sp. strain BR1 was incubated in AUM with (i) no SMX (open circles), (ii) with 1 mM SMX starting concentration (black filled circles) and (iii) with 1 mM SMX starting concentration, and daily adjustment of the SMX concentration to 1 mM (grey filled circles). A comparison of all three setups is depicted in plot (**A**). Plot (**B**) shows the OD_600_ measurements of setup ii alone, including the SMX concentration (**x**) which was determined photometrically. The growth of the setup (iii) is depicted in plot (**C**), including the daily measured SMX concentration (**x**) and the SMX adjustment points (**blue asterisk**). Plot (**D**) compares the OD_600_ measurements of setup (iii) to the current SMX degradation rate in [µmol mg_DW_^−1^ h^−1^].

Sulfonamide antibiotics, such as SMX, are used for the treatment of urinary tract infections and indeed several *Microbacterium* and *Arthrobacter* species have already been isolated from urine^47,48^. Moreover, both genera *Microbacterium* and *Arthrobacter* have been previously defined as medically relevant^49^, even though their known pathogenic potential is presumably rather low^47,48^. However, the implications of sulfonamide metabolizing coryneform bacteria must not be underestimated. Both *Arthrobacter* and *Microbacterium* have shown extreme resistance to UV sterilization, leading to their isolation even from clean rooms of a pharmaceutical production and NASA experimentation sites, respectively^50,51^. We were able to prove, that *Microbacterium* sp. strain BR1 is able to grow in artificial urine medium, in which it is even able to degrade SMX even when the SMX concentration of 1 mM was adjusted daily (Fig. 6). The presence of SMX in artificial urine medium did not lead to biomass increase of *Microbacterium* sp. strain BR1 cultures, but the antibiotic concentration was drastically decreased in such incubations. It has been shown that 3-amino-5-methylisoxazole, the dead end metabolite of SMX degradation in *Microbacterium* sp. strain BR1 lacks antibiotic activity^52^. Thus, the capability of *Microbacterium* sp. strain BR1 to degrade SMX even in urine containing readily degradable carbon sources may clear the way for infections by sulfonamide sensitive pathogens.

We found 16 resistance genes in *Microbacterium* sp. strain BR1 by querying the Comprehensive Antibiotic Resistance Database^53^ (Supplementary Table S2). Among them was the sulfonamide resistance gene *sul1*, encoding a mutated dihydropteroate synthase (DHPS) insensitive to inhibition by sulfonamides.

Overall, we identified catabolic genes enabling microbial growth on sulfonamides. Our experiments corroborate that bacteria can subsist on antibiotics^33^ and definitively resolve the controversy in the literature about the ability of microorganisms to use antibiotics as carbon and energy sources. The *sad* cluster is not related to any of the sulfonamide resistance genes known so far. It is furthermore remote from *sul1* on the chromosome of *Microbacterium*, includes a relaxase which suggests mobility (present or historical) and is present in several bacteria. Therefore we postulate that SadA should be considered as a part of the resistome, as it shows a high potential to evolve into an effective resistance gene. Moreover, strains able to subsist on antibiotics and thereby lowering the antibiotic concentration, have a fitness advantage over the ones having only traditional resistance genes, such as *sul1*. Therefore this cluster also bears the potential to contribute to accelerate the spreading of classic resistance mechanisms (*i.e*. here *sul1*). A corollary question is whether and how classic antimicrobial resistances affect the metabolic capacity of microorganisms, especially in terms of selection pressure, for promiscuous enzymes are often involved in co-metabolic and metabolic degradation processes. Since worldwide actions are taken to combat the spread of antibiotic resistance, knowledge on such molecular determinants is crucial and should become integral part of resistome studies and antibiotic resistance screenings.

## Materials and Methods

### Bioinformatic analyses

#### Phylogenetic tree of sulfonamide degrading bacterial isolates

The sequences of 16S rRNA genes were used to build a phylogenetic tree of sulfonamide degrading bacteria. Ugene V1.26^54^ was used as the graphical user interface for the bioinformatic tools. All sequences were aligned with MAFFT (advanced options were inactivated). The tree was built with PhyML Maximum Likelihood (Substitution model HKY85, equilibrium frequencies: optimized, number of substitution rate categories: 4, fast likelihood-based method: aLRT, tree improvement: SRT & NNI) and plotted with Ugene.

#### Genome sequencing

Genomic DNA of *Microbacterium* sp. strain BR1 was isolated from an overnight liquid culture using the DNeasy blood and tissue kit (Qiagen). Sequencing was performed on a Roche 454 platform using both a 3 kb paired end and a shotgun sequencing approach. Prediction of CDS was done by Critica, Glimmer and Prodigal. All predictions were merged together into one unique set of CDS. Additional annotation was performed using the RAST server^55,56^. The draft genome has been deposited at NCBI under BioProject number PRJNA394223.

The genomic DNA of *Microbacterium lacus* strain SDZm4 (DSM 26765) was isolated with the GenElute Bacterial Genomic DNA Kit (Sigma-Aldrich) from a 48 h old culture. The kit Promega QuantiFluor was used for quantification of the concentration of genomic DNA and of the sequencing library. We used the KAPA HyperPlus Kit for enzymatic fragmentation and whole library preparation, performing size selection step with NucleoMag magnetic beads (Macherey Nagel). TruSeq DNA PCR-Free LT Library adapters were used. Libraries were sequenced with the MiSeq Reagent Kit v2 (500 cycles) on the Illumina MiSeq system. The resulting paired reads were further analysed with the MyPro pipeline for genome assembly and annotation^57^. The draft genome has been deposited at NCBI under BioProject number PRJNA394221.

#### CARD analysis of *Microbacterium* sp. strain BR1’s genome

The Comprehensive Antibiotic Resistance Database (CARD) analysis^53^ of the 10 genome contigs of *Microbacterium* sp. strain BR1 was carried out on February 14^th^ 2017. The genome was screened with the Resistance Gene Identifier (RGI) for perfect and strict hits to known resistance genes.

#### Identification of *sad* genes in sulfonamide mineralizing isolates

Genomes of the strains *Microbacterium* sp. strain C448 (BioProject accession number PRJNA170195) and *Arthrobacter* sp. strain D2 (BioProject accession number PRJNA314012) and D4 (BioProject accession number PRJNA314014) were downloaded from National Center for Biotechnology Information (NCBI) repositories and analysed locally. Ugene V1.20.00 was used as the graphical user interface.

A local Blast+ database containing the amino acid sequences of the SadA, the SadB and SadC from *Microbacterium* sp. strain BR1 was built and queries were run against the genomes of all four genomes, including the one from *Microbacterium* sp. strain SDZm4. Applied settings were the following: search: blastx, expectation value: 10, best hits limit: 100, both strands, word size: 11, gap costs 2 2, match scores 1–3; X dropoff values: gapped alignment: 30 bits, ungapped extensions: 20 bits.

The identity of the *sad* gene cluster and its surrounding was carried out by making Blast+ databases of each genome and carrying out local Blast analysis against the genome of *Microbacterium* sp. strain BR1 and vice versa.

#### Alignment of *sad* genes and enzymes

Gene and enzyme sequences were aligned offline with MAFFT (advanced options were inactivated) in the graphical user interface Ugene V1.26^54^. The sequence of *Microbacterium* sp. strain BR1 was set as reference sequence to determine identities among the sequences. The phylogenetic tree of the SMX-MO was built with PhyML Maximum Likelihood (Substitution model HKY85, equilibrium frequencies: optimized, number of substitution rate categories: 4, fast likelihood-based method: aLRT, tree improvement: SRT & NNI) and plotted with Ugene.

### Molecular biology

#### Plasmid construction with *sad* genes

The genes *sadA* and *sadC* encoding for the SMX-monooxygenase and FMNR were codon optimized for *E. coli* and synthesized by MWG Operon (Ebersberg, Germany). All three *sad* genes were separately cloned into a pET28a vector, downstream of a hexahistidine tagged small ubiquitin related modifier (SUMO, Sequence 1) under control of a T7 promotor (plasmid construct provided by Ricardo Adaixo). A restriction free cloning strategy was used for the construction of all three plasmids. The primer design was carried out with the online tool of the website rf-cloning.org^58^. The retrieved primer sequences were synthesised by Eurofins (Table 1) and the PCRs was carried out accordingly (Tables 1, 2).

**Table 1:** Primers for RF-Cloning.

Construct Name	Forward/	Sequence [5′ - >3′]	Annealing Temp. [°C]	
	Reverse		Plasmid	Target
pET28a_Nt6HisSUMO-MOII_codon_opt	Forward	CTCACCTCGAACAGATTGGTGG-CATGAAATCTGTCCAAAGCGCT	61	56
	Reverse	GGTGGTGGTGGTGCTCGAGTCACTAAATCGG-CATGACGAACTC	64	55
pET28a_Nt6HisSUMO-FMNR	Forward	CTCACCTCGAACAGATTGGTGGCATGAC-CTCCGAATCACCAAC	61	56
	Reverse	GGTGGTGGTGGTGCTCGAG-TCATCAGATGATCGCGGAGCG	64	58
pET28a_Nt6HisSUMO-MOI	Forward	CTCACCTCGAACAGATTGGTGGCATGGTCGA-TAGCAGTTTGCC	61	56
	Reverse	GGTGGTGGTGGTGCTCGAGTCATCAAAC-CAGAGGCGTAACG	64	56

**Table 2:** RF-Cloning parameters for 2nd PCR.

Construct Name	Extension Time [min]	Insert [ng]	Plasmid [ng]
pET28a_Nt6HisSUMO-MOII_codon_opt	2:14	309	70
pET28a_Nt6HisSUMO-FMNR	2:01	151.2	69.9
pET28a_Nt6HisSUMO-MOI	2:13	298.8	69.9

*E. coli* XL10Gold competent cells were transformed with the construct and colonies were checked by colony PCR using T7 primer. The plasmids were purified from positive clones and sequenced (MWG Operon). Finally, competent *E*. *coli* Arctic Express were transformed with the plasmid constructs.

Sequence 1: SUMO-tag – Supports proper folding of heterologously expressed enzymes

TCTGACTCCGAAGTCAATCAAGAAGCTAAGCCAGAGGTCAAGCCAGAAGTCAAGCCTGAGACTCACATCAATTTAAAGGTGTCCGATGGATCTTCAGAGATCTTCTTCAAGATCAAAAAGACCACTCCTTTAAGAAGGCTGATGGAAGCGTTCGCTAAAAGACAGGGTAAGGAAATGGACTCCTTAAGATTCTTGTACGACGGTATTAGAATTCAAGCTGATCAGACCCCTGAAGATTTGGACATGGAGGATAACGATATTATTGAGGCTCACCTCGAACAGATTGGTGGC.

#### DNA clean-up

DNA was cleaned after PCR with the “NucleoSpin Gel and PCR Clean-up” kit (Macherey-Nagel, Düren, Germany) and the peqGOLD Cycle-Pure kit (VWR International AG, Zürich, Schweiz), respectively.

#### Plasmid purification

Plasmids from *E. coli* XL10-Gold (Agilent) cells were extracted and purified with the “NucleoSpin Plasmid” kit (Macherey-Nagel).

#### Preparation of chemically competent *E. coli* cells

100 ml of a fresh *E. coli* XL10Gold culture in LB medium with an OD_600_ between 0.5 and 0.7 were chilled on ice for 15 min before centrifugation with 4′500 × g at 4 °C for 5 min. The supernatant was discarded and the cells were gently suspended in 40 ml TFBI buffer (30 mM sodium acetate, 50 mM MgCl2, 100 mM NaCl, 15% (w/v) glycerol, pH 6.0; sterilized by filtration) by pipetting. After incubation on ice for 15 min and centrifugation with 4′500 × g at 4 °C for 5 min, the supernatant was discarded again and cells were suspended gently in 40 ml TFBII buffer (10 mM MOPS, 75 mM CaCl2, 10 mM NaCl, 15% (w/v) glycerol, pH 7.0; sterilized by filtration) by pipetting. The suspended cells were cooled on ice for 15 min before aliquots were stored at −80 °C.

#### Transformation of competent *E. coli* cells

To 50 µl of competent *E. coli* cells in a sterile 2 ml reaction tube, ca. 50 ng of plasmid DNA ( <  = 0.5 µl) were pipetted. The mixture was incubated on ice for 20 min. The tube was regularly and gently inverted during the incubation. A heat shock was carried out at 42 °C for 30 sec in a prewarmed Thermomixer (Eppendorf, Schönenbuch, Switzerland), before the incubation on ice was continued for additional 30 min. 250 µl of sterile SOC medium (20 g/l tryptone, 5 g/l yeast extract, 4.8 g/l MgSO4, 3.6 g/l dextrose, 0.5 g/l NaCl, 0.19 g/l KCl, adapted from Sigma-Aldrich #S1797) (RT) was added to the *E. coli* suspension, followed by an incubation at 37 °C with vigorous shaking on a Thermomixer for 1 h. 5 µl and 45 µl were plated on LB plates containing kanamycin (50 mg/l). The plates were incubated overnight at 37 °C.

### Microbiology

#### Acclimatization of *Microbacterium* sp. strain BR1

Four media composed of 0.5 mM SMX and different concentrations of Standard I medium were tested for optimizing the preparation of acclimatized *Microbacterium* cells. Therefore, 125 µl of a sterile 200 mM SMX stock in MeOH were pipetted into an autoclaved 250 ml Erlenmeyer flask under a laminar flow bench. As soon as the MeOH was evaporated 50 ml of the medium were added. The flasks were placed on a rotary shaker until the SMX was completely dissolved. Afterwards 25 µl of a *Microbacterium* glycerol stock were added to the biotic flasks. The experiment was carried out in duplicates. The OD was measured at 600 nm and the SMX concentration was analyzed by HPLC.

#### *Cultivation* of the expression strain *E. coli* Arctic Express

An overnight culture of *E. coli* Arctic Express (*E. coli* AE) (Agilent) was grown at 30 °C in 20 ml LB-medium with 50 mg/l kanamycin (LB-Kan). 200 ml of the autoinduction medium ZYM-5052 in 1 l Erlenmeyer flasks were inoculated with the overnight culture to have a starting OD_600_ of 0.05. This culture was incubated for 24 h at 30 °C and 220 rpm. Cells were harvested by centrifugation with 4′500 × g at 4 °C for 15 min and resuspended in 50 mM PBS pH 7.0.

#### *Degradation* of ^14^C-labelled SMX by *E. coli* mutants

An overnight *E. coli* AE culture containing either of the *sad* genes, was grown at 37 °C in 20 ml LB medium with Kanamycin (50 mg/l). 20 ml autoinduction medium ZYM-5052 was inoculated with the overnight culture to have a starting OD_600_ of 0.05 and the cultures were incubated at 37 °C. As soon as the functional expression of the MO1 was visible, because blue indigo pellets started to form, a final concentration of 100 µM ^14^C-labelled SMX (0.85 kBq ml^−1^) was added to the cultures, the temperature was decreased to 23 °C and the cultures were incubated overnight. Samples were centrifuged with 16000 × g and 4 °C for 15 min and then filtered with 0.45 µm PVDF filter syringe filters. Those samples were analysed by LC-DAD coupled to a LSC detector (LC-DAD-LSC). All setups were carried out in duplicates. Abiotic controls consisted only of the medium and ^14^C-SMX. *E. coli* controls contained in untransformed *E. coli* Arctic Express cells.

#### Detection *of*^14^C-labelled paracetamol in *E. coli* cultures

*E. coli* AE *sadA* were concentrated to a calculated OD_600_ of 11. 12 ml were transferred to a 50 ml centrifugation tube. ^14^C-SMX was added to a final concentration of 470 µM (corresponding to 4.23 kBq ml^−1^). The abiotic and biotic control were missing the *E. coli* AE *sadA* and the ^14^C-SMX, respectively. The mixtures were incubated on a rotary shaker with 230 rpm and RT. All setups were carried out in duplicates and 2 ml samples were taken every hour. The samples were centrifuged with 21′500 × *g*, at 4 °C for 15 min. The supernatant was filtered with 0.45 µm PVDF filters. 1 ml was directly used for HPLC analysis and 900 µl were derivatized with 100 µl acetic anhydride, incubated at 30 °C for 2 h and neutralized with 235 µl 10 M NaOH, before LC-DAD-LSC and LC-MS analysis. The LC-DAD-LSC and LC-MS analyses were carried out as described previously^20^.

#### *Degradation* of 4-aminophenol by *E. coli* AE *sadB*

2 ml of *E. coli* AE and *E. coli* AE *sadB* resting cells with a calculated OD_600_ of 20 were transferred to 50 ml centrifugation tubes, each. 2 ml of a 400 µM 4-aminophenol stock solution was added. The abiotic control contained only PBS buffer and 4-aminophenol. All setups were carried out in triplicates and incubated on a rotary shaker with 230 rpm at RT. 750 µl samples were taken every hour and centrifuged with 21′500 × g, at 4 °C for 15 min. 500 µl of the supernatant was transferred to a fresh 1.5 centrifugation vial and 50 µl acetic anhydride were added before the mixture was incubated at 30 °C for 1 h. Samples were neutralized by the respective addition of 115 µl 10 M NaOH before they were analysed by LC-DAD (compare SI).

#### *Cultivation* of *Microbacterium* sp. strain BR1 in mineral medium

Acclimatized *Microbacterium* sp. strain BR1 cultures were used as inoculum for cultivations in mineral medium. MMO medium as described by Stanier *et al*.^59^ was prepared and amended with 0.25% of Schlegel’s vitamin solution containing 10 mg/l *p*-aminobenzoate, 2 mg/l of biotin, 20 mg/l of nicotinic acid, 10 mg/l of thiamine-HCl, 5 mg/l of Ca-panthothenate, 50 mg/l of pyridoxamine and 20 mg/l of vitamin B_12_.

#### Cultivation of *Microbacterium* sp. strain BR1 in artificial urine

Acclimatized *Microbacterium* sp. strain BR1 cultures were used as inoculum for artificial urine medium (AUM). The buffer strength of the artificial urine medium from Brooks and Keevil^60^ was increased from 14 to 50 mM to avoid Mg^2+^ and Ca^2+^ precipitation. For setups containing a final concentration of 1 mM SMX, dd H_2_O with 1 mM SMX was autoclaved to solubilize SMX. This 1 mM SMX H_2_O was used to solubilize the AUM ingredients instead of dd H_2_O. Both media were sterilized by filtration through 0.22 µm filter.

Four different setups were carried out: (i) an abiotic control consisting only of AUM, (ii) *Microbacterium* sp. strain BR1 in AUM, (iii) *Microbacterium* sp. strain BR1 in AUM with 1 mM SMX starting concentration, (iv) *Microbacterium* sp. strain BR1 in AUM with 1 mM SMX starting concentration. In setup (iv) the SMX concentration was adjusted to 1 mM every 24 h. All setups were carried out in triplicates. The cultures and controls were incubated on a rotary shaker with 130 rpm and 28 °C. 0.5 ml samples were taken daily and the biomass rise was estimated by OD_600_ measurements. The SMX concentration was determined photometrically^21^.

## Electronic supplementary material

Supplementary information
