# Supplementary material for: FMNH2-dependent monooxygenases initiate catabolism of sulfonamides in Microbacterium sp. strain BR1 subsisting on sulfonamide antibiotics
Source: Sci Rep. 2017 Nov 17;7:15783. doi: 10.1038/s41598-017-16132-8 (PMC5693940; doi:10.1038/s41598-017-16132-8)
Supplement: Supplementary file 1 — Supplementary information [file 41598_2017_16132_MOESM1_ESM.pdf]

$FMNH_2$-dependent monooxygenases initiate catabolism of sulfonamides in *Microbacterium* sp. strain BR1 subsisting on sulfonamide antibiotics

Benjamin Ricken, Boris Kolvenbach, Christian Bergesch, Dirk Benndorf, Kevin Kroll, Hynek Strnad, Cestmir Vlcek, Ricardo Adaixo, Frederik Hammes, Patrick Shahgaldian, Andreas Schäffer, Hans-Peter E. Kohler, Philippe F.-X. Corvini

SI Results

Table S 1: Literature overview on mineralization studies with ^{14}C-labelled antibiotics. It has to be noted, that only defined parts of the antibiotic molecules were labelled with ^{14}C. It cannot be ruled out that mineralization rates for non-labelled moieties of the antibiotic were larger than the here stated rates.

**First author**	**Year**	**^{14}C-labelled antibiotic**	**Biomass**	**Mineralization observed**	**Reference**
Marengo	1996	Sarafloxazin hydrochloride	Different soils	< 0.6 %	1
Junker	2006	Benzylpenicillin, ceftriaxone and trimethoprim	Activated sludge	yes (only benzylpenicillin)	2
Wehrhan	2006	Sulfadiazine	Soil	0.3 % after 42 days	3
Schmidt	2008	Sulfadiazine	Soil	< 2 % after 218 days	4
Henderson	2008	Sulfamethazine	Small pond water microcosms	< 3 %	5
Bouju	2012	Sulfamethoxazole	5 bacterial Isolates	Yes	6
Islas-Espinoza	2012	Sulfamethazine	15 bacterial Isolates	Yes	7
Junge	2012	Difloxacin	Pig manure	< 0.2 % after 56 days	8
Topp	2013	Sulfamethazine	1 bacterial isolate	Yes	9
Tappe	2013	Sulfadiazine	1 bacterial isolate	Yes	10
Jessick	2013	Erythromycin	Sediment and manure	Yes	11
Reis	2014	Sulfamethoxazole	1 bacterial isolate	Yes	12
Kim	2004	Erythromycin A	Aquaculture sediment	Yes	13
Topp	2016	Erythromycin, clarithromycin	Soil	Yes	14

Distribution of radioactivity after incubation of *Microbacterium* with ^{14}C-SMX

The distribution of radioactivity was determined in an incubation of *Microbacterium* cells with ^{14}C-SMX after 5 days when 48 % of the initially applied amount of radioactivity was found in the CO_2 trap of the

setup (Figure S 1). The cell dry weight after 5 days was 4.1 mg ml^{-1}. 41 % of the initially applied ^{14}C were detected in the cell suspension and 1 % was recovered by washing the flask with EtOH. 28.7 % of the initially applied ^{14}C was bioavailable, as it can be dissolved by washing the cell pellet with dd H_2O. 3.9 % could be extracted from the biomass with 125 mM NaOH. 9.33 % was not extractable from the biomass and was recovered after combustion of the cell pellet. The overall recovered amount of radioactivity was 90 %. 10 % of ^{14}C could not be recovered.

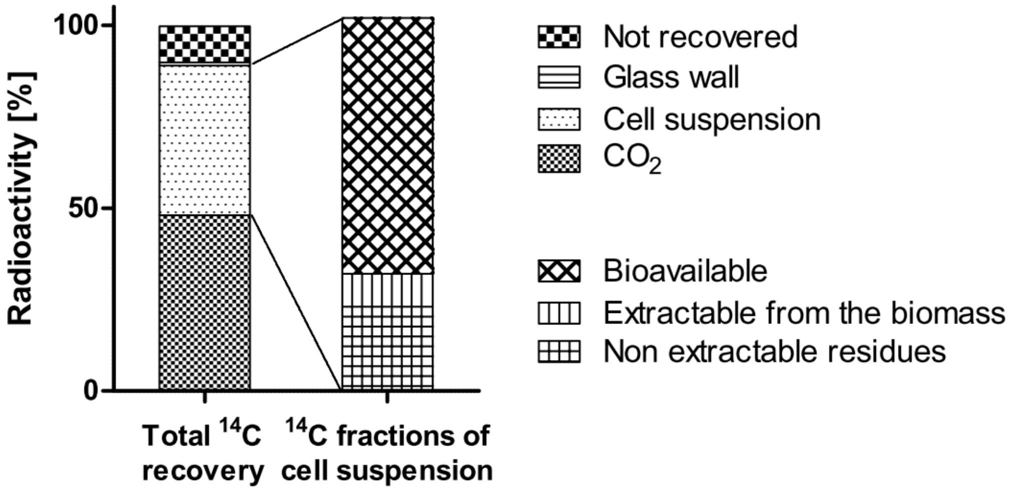

Figure S 1: ^{14}C recovery after a mineralization experiment After 5 days of incubation of Microbacterium with ^{14}C-SMX, the amount of ^{14}C was determined in the biomass, the cell suspension and at the inner flask glass wall. The ^{14}C amount in the medium was further fractionated in ^{14}C associated to the biomass, extractable from the biomass and bioavailable.

Table S 2: Resistance genes identified in Microbacterium sp. strain BR1 with CARD

Best_Hit_ARC	CUT_OF	Best_Hit_evalue	Best_Identites	AR0_category
lrfA	Strict	5.95E-153	57	efflux pump conferring antibiotic resistance
tet43	Strict	1.88E-82	40	efflux pump conferring antibiotic resistance
mfd	Strict	0	36	antibiotic target protection protein; fluoroquinolone resistance protein
TaeA	Strict	1.21E-106	35	efflux pump conferring antibiotic resistance
katG	Strict	0	63	antibiotic resistant gene variant or mutant; isoniazid resistance protein
EF-Tu	Strict	0	75	antibiotic resistant gene variant or mutant; elfamycin resistance protein; gene involved in self-resistance to antibiotic
TaeA	Strict	1.68E-113	39	efflux pump conferring antibiotic resistance
parY	Strict	0	65	aminocoumarin resistance protein; antibiotic resistant gene variant or mutant; gene involved in self-resistance to antibiotic
novA	Strict	1.57E-176	47	efflux pump conferring antibiotic resistance
alaS	Strict	0	39	aminocoumarin resistance protein
ileS	Strict	0	53	antibiotic resistant gene variant or mutant; mupirocin resistance protein
lmrB	Strict	6.52E-109	39	efflux pump conferring antibiotic resistance
TetB	Strict	7.47E-148	78	efflux pump conferring antibiotic resistance
murA	Strict	1.67E-100	42	fosfomycin resistance protein
tetA	Strict	6.60E-160	73	efflux pump conferring antibiotic resistance
sul1	Strict	0	100	antibiotic target replacement protein; sulfonamide resistance protein

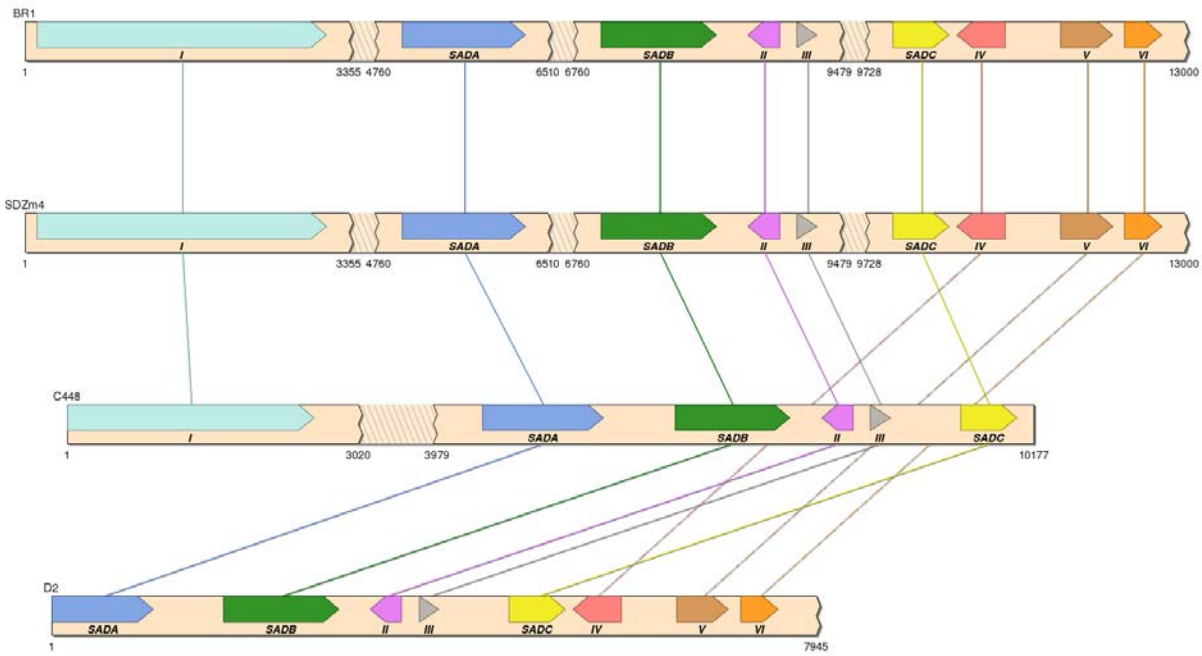

Figure S 2: Alignment of the sad cluster of Microbacterium sp. strain BR1 with homolog sequences found in the genomes of Microbacterium sp. strain C448, Microbacterium sp. strain SDZm4 and Arthrobacter sp. strain D2. Graph was created with SimpleSynteny[15].

The degradation of sulfonamides was tested in the presence of alternative sources of carbon in order to find ideal conditions for comparative proteome analysis. *Microbacterium* sp. strain BR1 was incubated with 480 µM SMX in different concentrations of complex medium. The results showed that the richer the nutritional conditions were, the slower was the catabolism of the antibiotic (Figure S 3).

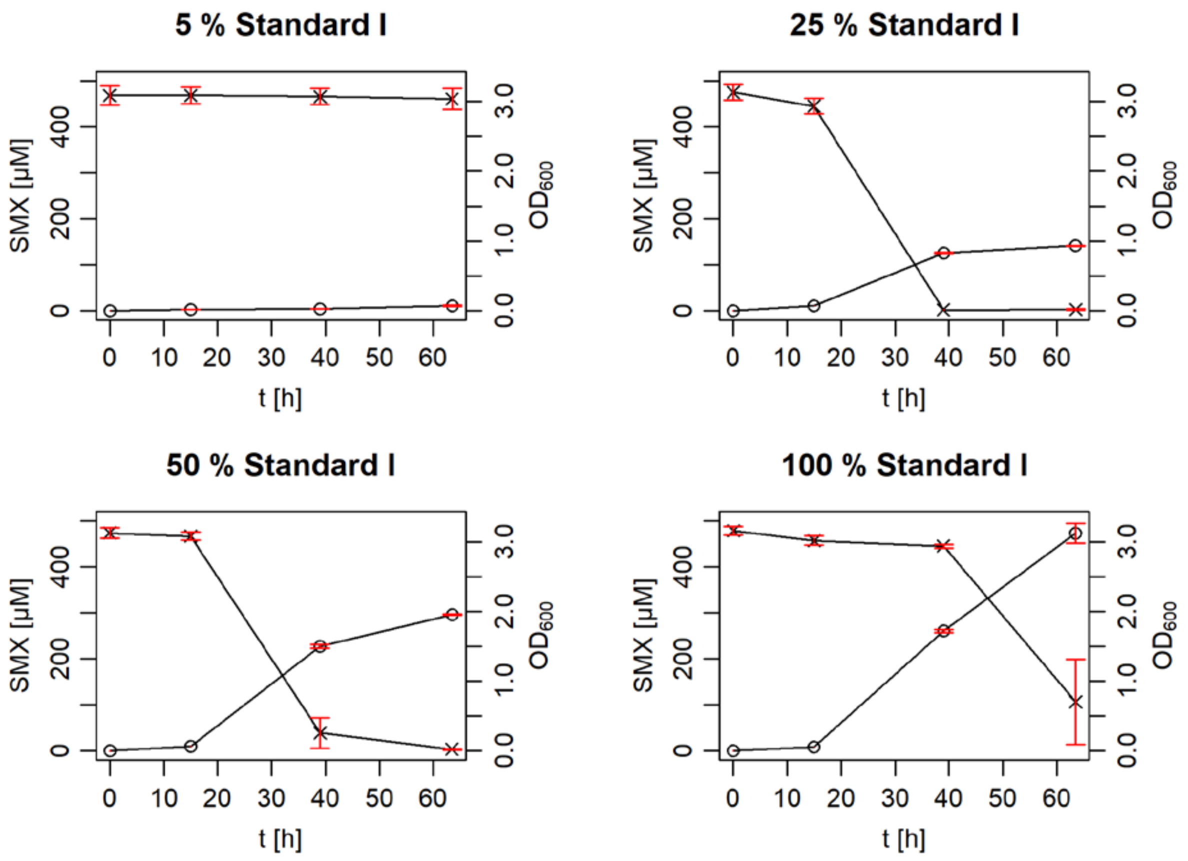

Figure S 3: Degradation of SMX by *Microbacterium* sp. strain BR1 in different concentrations of complex medium Standard I. Open circles: SMX concentration. x: *Microbacterium* sp. strain BR1 biomass measured as turbidity at 600 nm. Red error bars indicate the standard deviation of the mean (n=2).

This suggested a negative effect of the nutrient contained in the medium on the expression of the sulfonamide degradative genes. However, SMX assimilation by *Microbacterium* sp. strain BR1 was satisfactorily induced when the cells were incubated in dilute complex medium (25% Standard I medium containing 1 mM succinate) with 1 mM SMX.

Table S 3: Proteomic analysis of acclimatized and non-acclimatized Microbacterium sp. strain BR1 cells (compare bands marked in Fig. 3). Protein identification was based on BLAST search results with a Microbacterium sp. strain BR1 genome database (Microbacterium Accession) and on MASCOT search of NCBInr database (Protein* [Accession NCBInr]). MASCOT hits with sequence coverage(S[%]) higher than 40% are highlighted. Also shown are calculated molecular weight (MW**[kDa]) and calculated isoelectric point (pI).

Band	Microbacterium Accession	Protein* [Accession NCBInr]	Spectral Count	SC [%]	Mascot Score	MW** [kDa]	pI**
4	cdid1583	glucan phosphorylase [YP_001222140.1]	17	28.4	813.7	91.4	4.9
	cdid2412	phosphoketolase [WP_005049602.1]	13	19.5	494.3	90.1	5.3
	cdid2139	glycosyl transferase [WP_005051233.1]	11	15.9	507.1	95.0	5.2
	cdid453	isocitrate dehydrogenase [WP_005047978.1]	7	16.3	187.0	79.5	4.9
5	cdid277	elongation factor G [YP_004223802.1]	16	27.4	605.3	77.7	4.9
	cdid1485	transketolase [YP_004226495.1]	15	20.2	732.5	93.1	5.3
	cdid2931	DNA gyrase subunit B [YP_004224348.1]	11	11.0	272.3	74.9	4.9
6	cdid2467	dehydrogenase subunit E1 [WP_007735872.1]	41	49.1	1856.8	77.9	5.0
	cdid2154	phosphotransacetylase [YP_004225468.1]	6	8.2	243.8	83.6	5.5
	cdid2667	dehydrogenase [WP_021809426.1]	1	1.7	229.8	78.1	4.9
7	cdid734	ATP synthase subunit alpha [WP_005048638.1]	27	37.9	1160.9	58.3	4.7
8	cdid2026	chaperone GroEL [WP_005051072.1]	37	59.0	2035.9	57.0	4.7
	cdid2468	dehydrogenase subunit E2 [WP_021809427.1]	18	33.5	726.1	46.1	4.6
	cdid1508	30S ribosomal protein S1 [YP_004222847.1]	16	26.6	865.7	52.8	4.7
9	cdid2690	oxidoreductase [WP_009478410.1], indole oxygenase [YP_709118.1]	30	55.9	1611.9	44.7	4.7
	cdid2469	succinate-semialdehyde dehydrogenase [YP_003339583.1]	12	36.4	502.2	48.6	4.8
10	cdid2689	oxidoreductase [YP_002782537.1], indole oxygenase [WP_009081741.1]	38	70.4	2027.2	43.2	4.8
	cdid2044	sugar ABC transporter [WP_018172581.1]	9	26.7	345.6	39.3	5.4
12	cdid1971	uridylyltransferase [WP_005052222.1]	8	21.1	399.0	34.8	4.8
	cdid659	RNA polymerase subunit [WP_019182252.1]	8	26.7	268.1	24.3	5.4
	cdid2682	oxidoreductase [WP_016887960.1]	6	33.4	339.6	29.7	6.0
13	cdid2583	dehydrogenase/reductase [WP_003938938.1]	12	34.7	375.7	29.5	4.7
	cdid296	30S ribosomal protein S5 [WP_005050500.1]	7	15.1	391.4	24.2	10.5
	cdid1305	elongation factor Ts [YP_004226119.1]	4	20.7	310.2	29.3	4.8
	cdid2586	dehydrogenase/reductase [YP_001072314.1]	4	17.3	196.0	28.4	5.1

Band	Microbacterium Accession	Protein* [Accession NCBInr]	Spectral Count	SC [%]	Mascot Score	MW** [kDa]	pI**
14	cdid2686	enoyl-CoA hydratase [WP_005264228.1]	9	28.6	537.1	23.2	4.4
	cdid2681	dehydrogenase/reductase [WP_003996216.1]	2	9.3	91.1	25.9	6.1
15	cdid1496	superoxide dismutase [YP_004226506.1]	5	12.0	154.2	23.2	5.1
	cdid1303	ribosome recycling factor [WP_005050254.1]	2	16.3	110.4	20.4	5.3
	cdid2925	DNA/RNA-binding protein [YP_004224357.1]	1	6.4	53.1	18.9	4.3
16	cdid2687	flavin reductase-like domain protein [YP_003915889.1]	11	49.0	517.8	20.5	5.2
	cdid2466	glyoxalase/bleomycin resistance/dioxygenase [YP_925639.1]	6	25.6	175.6	20.2	4.7
17	cdid2935	DNA/RNA helicase [YP_004224343.1]	7	29.4	291.7	20.9	10.3
18	cdid350	co-chaperonin GroES [YP_004223656.1]	6	61.8	257.4	10.9	4.6
	cdid280	30S ribosomal protein S10 [WP_018171443.1]	3	37.3	92.3	11.5	10.0
	cdid2684	endoribonuclease L-PSP [YP_004963288.1]	2	21.9	168.2	13.7	4.4

Sequential purification of SadA

In order to purify the SMX degrading monooxygenase SadA from crude cell extract of *Microbacterium* sp. strain BR1, sequential purification was performed by means a weak anion exchange column (DEAE), followed by a strong anion exchange column (Mono Q). The activity of every fraction was determined with the NADH assay and degradation of SMX was verified by HPLC measurements. Early purification attempts revealed that upon purification beyond the DEAE column, activity was irreversibly lost, which we tentatively attributed to the lack of a reductase as a component in this catalysis. In order to verify this hypothesis, both a commercially available FMN reductase from *E. coli* (FRE) and FMN were added to fractions of a Mono Q-fractioned crude cell extract (without prior DEAE fractionation). The NADH activity assay revealed, that only in the presence of FRE the sulfonamide monooxygenase was active (Figure S 4), which was also verified by quantification of the remaining SMX by HPLC. An active fraction amended with 0.1 U of FRE degraded 80 µM in 30 min, whereas the same fraction without the addition of FRE degraded only 8 µM.

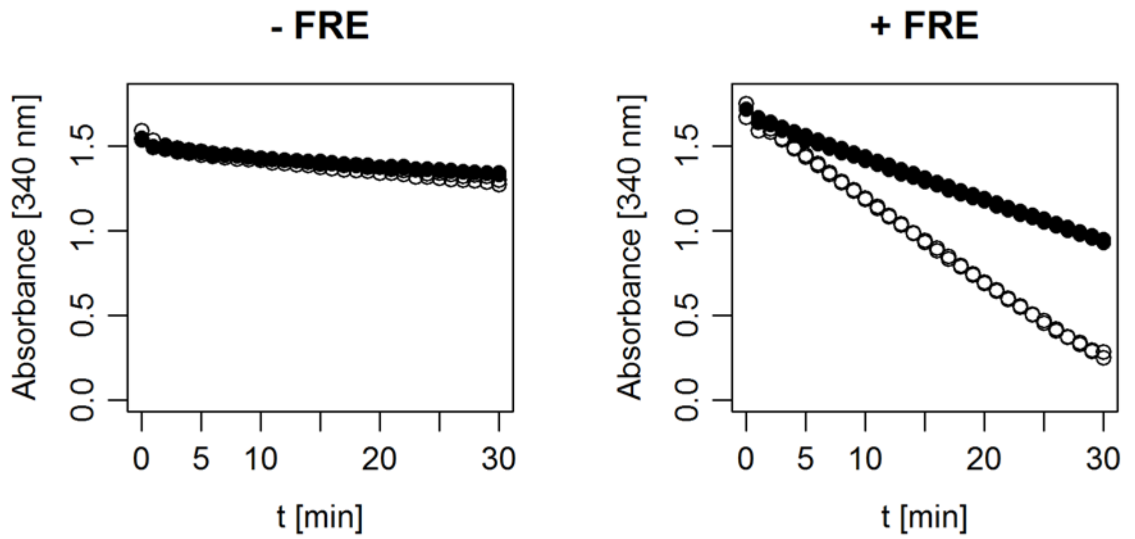

Figure S 4: NADH consumption of fractionized Microbacterium sp. strain BR1 cell extract with and without FMN reductase as determined by the NADH assay. Crude cell extract of *Microbacterium* sp. strain BR1 was fractionized by means of FPLC with strong anion exchange column Mono Q. Fractions were analysed with the NADH assay in the absence (- FRE) and presence (+ FRE) of a *E. coli* FMN reductase. Depicted are both experiments with the active fraction, bearing the enzyme responsible for SMX degradation. **Open circles** indicate the samples with SDZ and **filled circles** samples without SDZ.

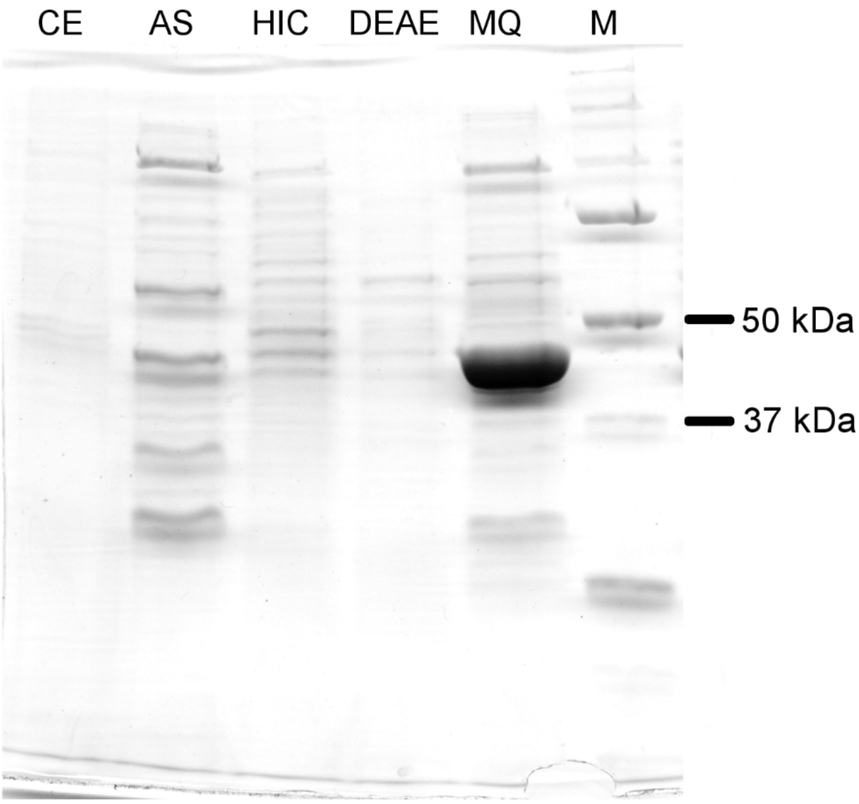

Figure S 5: SDS-PAGE of samples containing SadA after sequential purification steps. CE: crude cell extract (14 µg loaded); AS: Resuspended pellet after 70 % $(NH_4)_2SO_4$ precipitation (14 µg loaded); HIC: pool of active HIC fractions after desalting (3 µg loaded); DEAE: pool of active DEAE fractions after desalting (4 µg loaded); MQ: active fraction after Mono Q purification (13 µg loaded); M: 8 µl „Precision Plus Protein" BioRad. A detailed overview of the activities per fraction is shown in Table S 4.

Table S 4: Purification of the SMX-MO from *Microbacterium* sp. strain BR1. The activities for the SMX-MO were indirectly measured via NADH consumption rates.

	**Volume [ml]**	**Activity [U]**	**Protein [mg]**	**Spec. act. [U mg^{-1}]**	**Purification factor**	**Yield [%]**
Crude CE	40	2539	109.22	23	1	100
Pellet 70 %	6	3624	214.46	17	0.73	57
HIC (pool)	2	241	30.74	8	0.34	2
DEAE (pool)	2	102	8.80	12	0.50	1
Mono Q (1 fraction)	1	87	0.84	104	4.49	3

Identification of 4-aminophenol in incubations of *E. coli sadA*

HPLC-MS analyses of incubations of E. coli Arctic Express cells expressing sadA yielded 4-aminophenol, which could be verified by derivatizing samples and comparing the mass spectrum to authentic 4-aminophenol (Figure S 6).

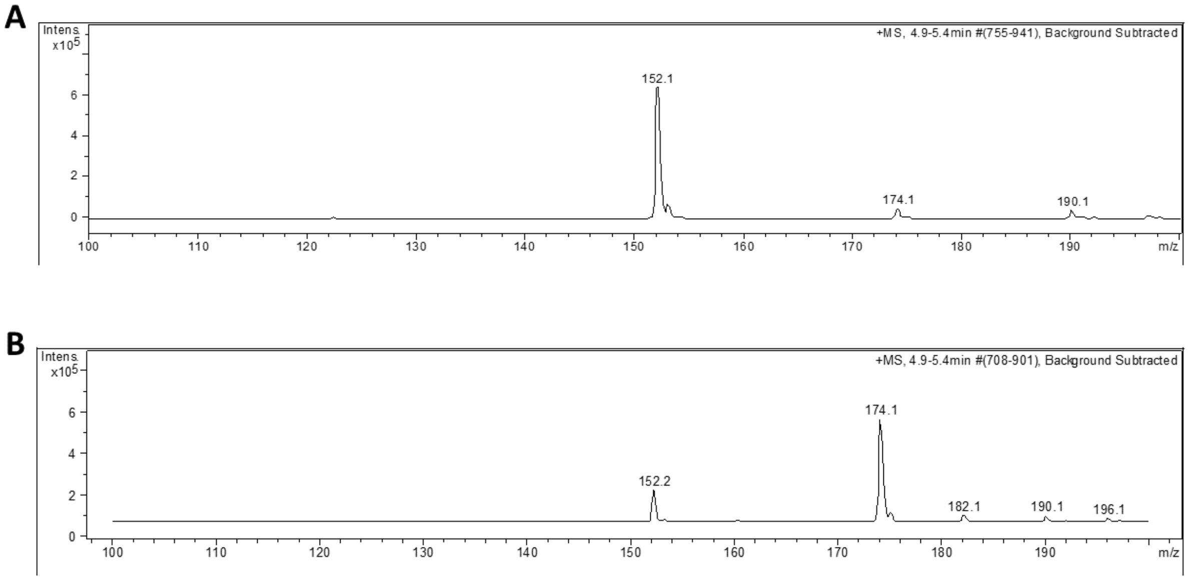

Figure S 6: HPLC mass spectra of samples after derivatization with acetic acid anhydride: authentic 4-aminophenol (A) and incubations of *E. coli sadA* (B). N-acetylated 4-aminophenol (molecular mass: 151.2 g/mol) measured in the positive mode features an m/z of 152, corresponding to the parent compound with a proton adduct, while m/z 174 corresponds to the sodium adduct ion.

Degradation studies of 4-aminophenol with *E. coli* AE *sadB*

Degradation studies of 4AP with *E. coli* AE *sadB* resting cells confirmed the hypothesis that the second monooxygenase is able to degrade 4AP. The 4AP concentration was measured by HPLC-DAD, after derivatization of 4AP with acetic anhydride (compare Figure S 7).

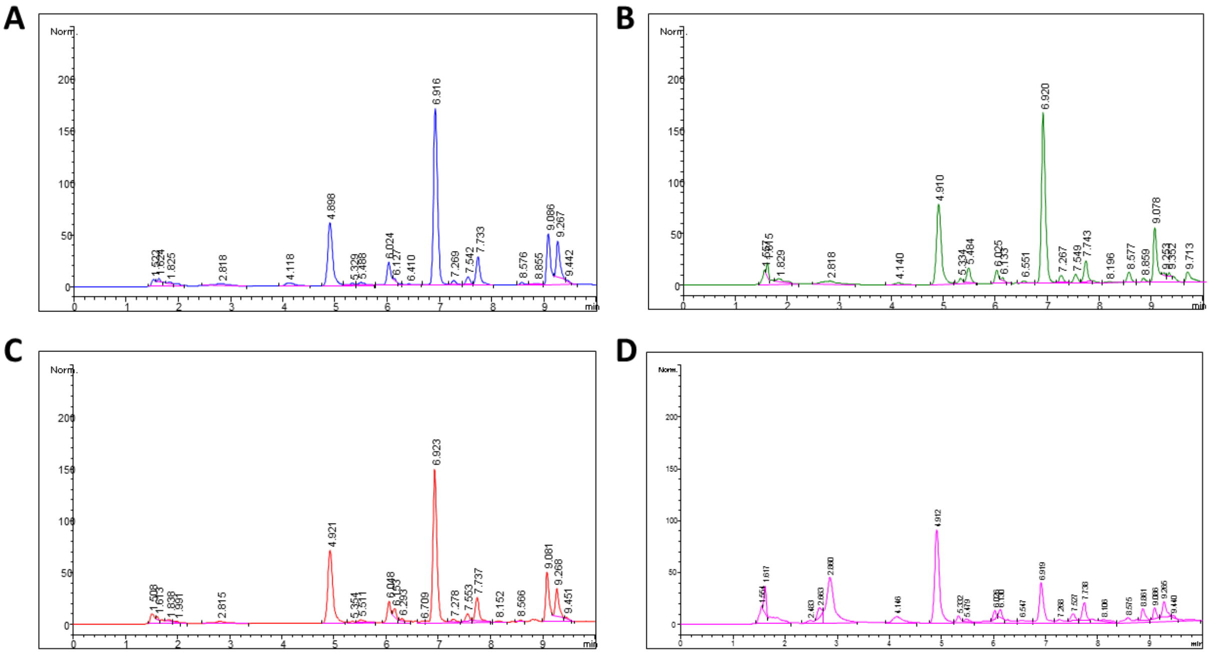

Figure S 7: HPLC chromatograms of N-acetylated 4-aminophenol (RT 6.9 min) detected in the supernatant of *E. coli* AE *sadB* during 4-aminophenol degradation. Shown are chromatograms of incubations of non-transformed cells of E. coli Arctic Express after 0 (A) and 120 (B) minutes of incubation with 4-aminophenol, respectively, as well as incubations of E. coli *sadB* after 0 (C) and 120 (D) minutes of incubation with 4-aminophenol, respectively.

Additionally, the formation of THB was non-quantitatively detected by GC-MS in the supernatant of *E. coli* AE *sadB* cultures (Figure S 8). 4AP was neither degraded by untransformed *E. coli* AE resting cells, nor in the buffer control.

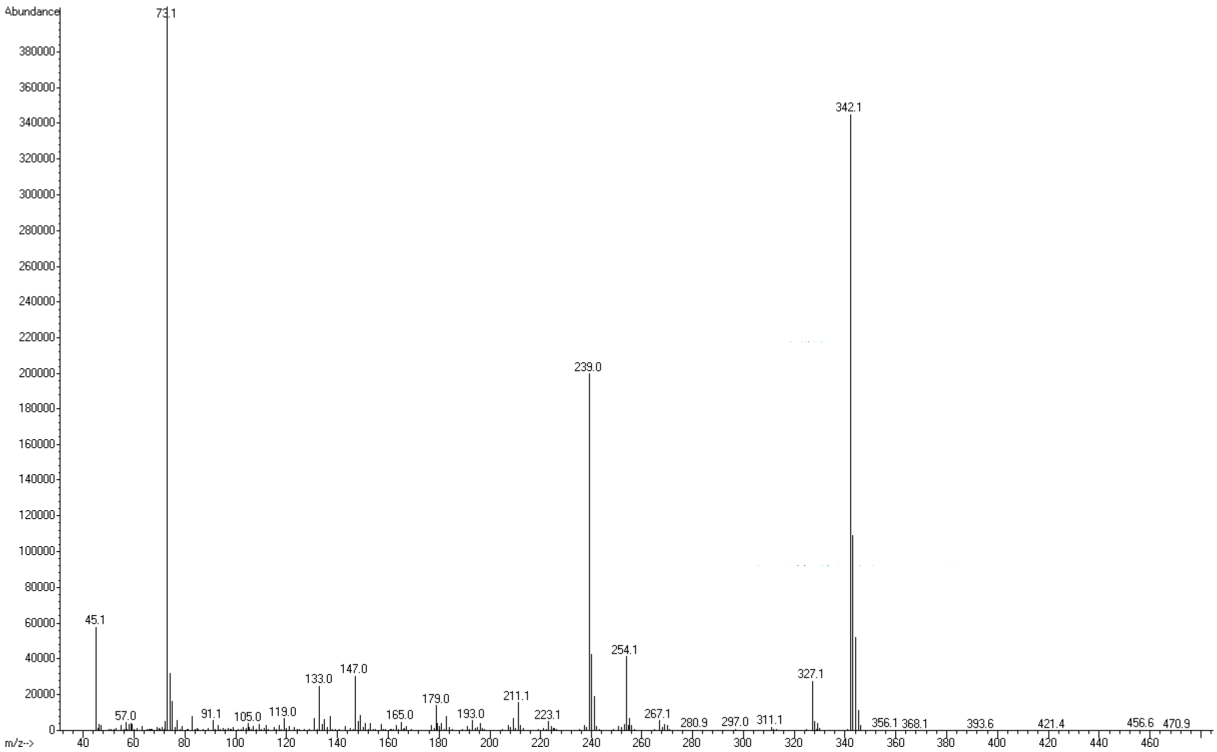

Figure S 8: GC-MS mass spectrum of THB detected in the supernatant of *E. coli* AE *sadB* during 4AP degradation.

Table S 5: Closest relatives of enzymes encoded by genes in the SMX-cluster

**Accession No.**	**Enzyme**	**Strain**
Phylogeny tree of SadA		
	SadA	*Microbacterium* sp. strain BR1
CDJ99310.1	Acyl-CoA dehydrogenase, C-terminal domain protein	*Microbacterium* sp. C448
OEH61722.1	hypothetical protein A5N17_13005	*Arthrobacter* sp. D2
OEH57813.1	hypothetical protein A5N17_22230	*Arthrobacter* sp. D2
OEH63558.1	hypothetical protein A5N13_14625	*Arthrobacter* sp. D4
WP_067118097.1	oxidoreductase	*Streptomyces yokosukanensis*
WP_073734107.1	oxidoreductase	*Streptomyces* sp. CB02488
WP_018102908.1	hypothetical protein	*Streptomyces*
WP_037951145.1	oxidoreductase	*Streptomyces* sp. PRh5
OKI93236.1	oxidoreductase	*Streptomyces* sp. CB01249
WP_065475849.1	oxidoreductase	*Streptomyces* sp. PTY087I2
pdb|3AFE|	3-Hsa Monooxygenase	*Mycobacterium tuberculosis*
pdb|2JBR|	4-Hydroxyphenylacetate Hydrox-ylase	*Acinetobacter baumanni*
pdb|2OR0|	Putative Hydroxylase	*Rhodococcus* sp. Rha1
pdb|2RFQ|	3-Hsa Hydroxylase	*Rhodococcus* sp. Rha1
Phylogeny tree for SadB		
	SadB	*Microbacterium* sp. strain BR1
CDJ99309.1	putative oxidoreductase	*Microbacterium* sp. C448
OEH60118.1	oxidoreductase	*Arthrobacter* sp. D2
WP_027935525.1	oxidoreductase	*Amycolatopsis* sp. ATCC 39116
WP_067161342.1	oxidoreductase	*Mycobacterium* sp. 1245805.9
WP_066939516.1	oxidoreductase	*Mycobacterium* sp. 1554424.7
SEF20031.1	Acyl-CoA dehydrogenase	*Amycolatopsis pretoriensis*
SFK75475.1	Acyl-CoA dehydrogenase	*Amycolatopsis sacchari*
WP_025350014.1	oxidoreductase	*Nocardia nova* SH22a
WP_072951565.1	oxidoreductase	*Rhodococcus koreensis*
WP_015889093.1	oxidoreductase	*Rhodococcus opacus* B4
pdb|3AFE|	3-Hsa Monooxygenase	*Mycobacterium tuberculosis*
pdb|2JBR|	4-Hydroxyphenylacetate Hydrox-ylase	*Acinetobacter baumanni*
pdb|2OR0|	Putative Hydroxylase	*Rhodococcus* Sp. Rha1
pdb|2RFQ|	3-Hsa Hydroxylase	*Rhodococcus* Sp. Rha1
Phylogeny tree for SadC		
	SadC	*Microbacterium* sp. strain BR1
WP_036299413.1	flavin oxidoreductase	*Microbacterium* sp. C448
WP_051513618.1	flavin oxidoreductase	*Arthrobacter* sp. D2
WP_031282619.1	flavin oxidoreductase	*Corynebacterium*-like bacterium B27
WP_066040788.1	flavin oxidoreductase	*Herbiconiux solani*
KUM29529.1	flavin oxidoreductase	*Arthrobacter* sp. EpRS66
WP_060700794.1	flavin oxidoreductase	*Arthrobacter halophytocola*
WP_082689349.1	flavin oxidoreductase	*Arthrobacter* sp. EpRS66
WP_047545568.1	flavin oxidoreductase	*Microbacterium* sp. CF335
WP_070348958.1	flavin oxidoreductase	*Arthrobacter* sp. SW1
WP_081638065.1	flavin oxidoreductase	*Arthrobacter* sp. PAO19
pdb|2QCK|	Flavin reductase domain protein	*Arthrobacter* Sp. Fb24
pdb|4L82|	Putative Oxidoreductase	*Rickettsia felis*

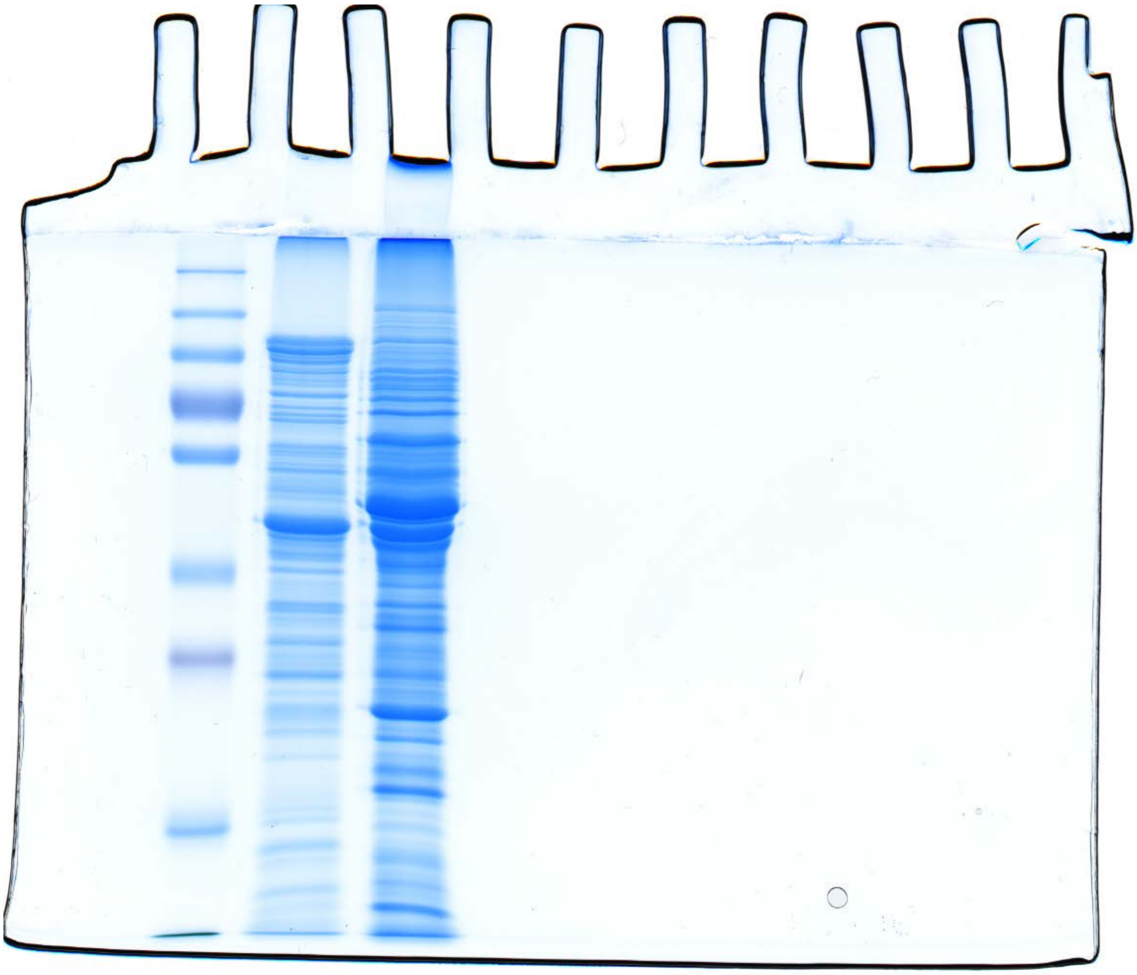

Figure S 9: original, uncropped image, from which Figure 2 has been obtained. No changes have been made to the image, merely the area of interest has been cropped.

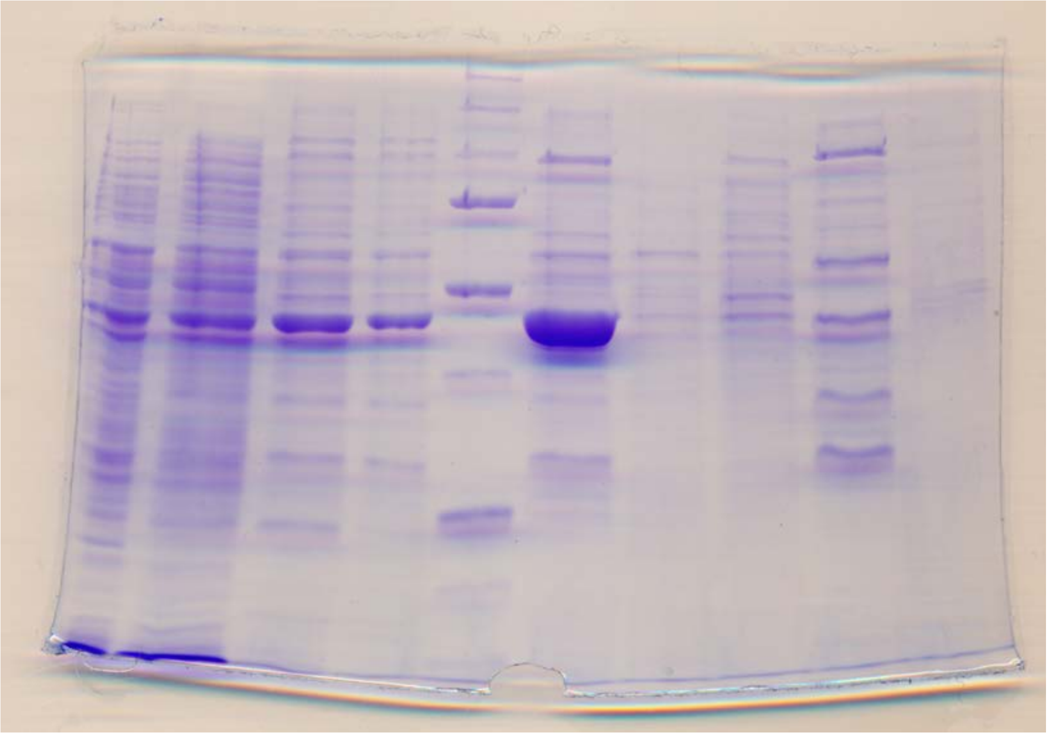

Figure S 10: original, uncropped image, from which Figure S 6 has been obtained. The image was flipped horizontally, converted to grayscale and cropped to the area of interest. The lanes, from left to right were loaded with the following samples: 15µL per lane of fractions 13, 12, 10 and 9, respectively, of the MonoQ fractionation step, 8 µl „Precision Plus Protein“ standard (BioRad); 15 µL of the active MonoQ fraction (fraction 11); 5 µl of a pool of active DEAE fractions after desalination; 3 µl of a pool of active HIC fractions after desalination; 1 µl of resuspended pellet after 70 % (NH4)2SO4 precipitation; 5 µl crude cell extract.

SI Materials & Methods

Determination of biomass associated radioactivity

After the last measurement point of ^{14}C-SMX mineralization experiments, 10 ml of the remaining cell suspension were used for the determination of the ^{14}C amount associated to the biomass and 5 ml were dried at 125°C for 2 h to determine the cell dry weight. The empty Erlenmeyer flask was washed with 10 ml EtOH and 1 ml was analyzed by LSC for the determination of ^{14}C-SMX adsorbed to the flask wall. The cell suspension was centrifuged at 4500 × *g* and 4°C for 5 min. 1 ml of the supernatant was measured by LSC and the remaining supernatant was discarded. The pellet was suspended in 10 ml dd H_2O and centri-

fuged again. 1 ml was taken for the LSC measurement and the remaining supernatant was discarded. The total amount of the recovered radioactivity in those fractions was designated as bioavailable ^{14}C. The pellet was then suspended in 5 ml 125 mM NaOH. This suspension was centrifuged as described above, 1 ml of the supernatant was analyzed by LSC and the remaining liquid was discarded. The last washing step was repeated four times. The biomass was combusted afterwards with a sample oxidizer. The formed CO_2 of the oxidized sample was trapped in Carbo Sorb E (PerkinElmer) before LSC analysis using Permafluor (PerkinElmer) as scintillation cocktail.

Analysis of protein extracts of *Microbacterium* sp. strain BR1 and protein identification

The proteins were extracted from biomass using phenol extraction and subsequent precipitation with organic solvents as described previously[16]. The precipitated proteins were resuspended in 2 mL of a solution containing 7 M urea, 4 M thiourea and 0.01 g mL^{-1} dithiothreitol. Protein concentrations were determined by a quantification protocol according to Schweikl *et al.*[17] using amido black dye. 25 µg of protein were precipitated with five volumes of ice-cold acetone. The pellet was solubilised in 11 µl of sample buffer for SDS-PAGE and separated on an acylamide gel (12% T, 3 % C) as described previously[18]. Bands of the Coomassie stained SDS–PAGE gel were cut and submitted to tryptic digestion[19]. Finally, extracts of a fragment were pooled, dried and stored at −20°C. Tryptic peptides were analyzed by nano-HPLC–MS/MS (amaZon™ ETD mass spectrometer nano electrospray ionization, Bruker Daltonik; coupled online with the UltiMate® 3000 RSLCnano splitless reverse phase liquid chromatography system, Thermo Fisher Scientific) as described previously. Database search with Mascot™ (version 2.2, Matrix Science)[20] was carried out against the genome of *Microbacterium* sp. strain BR1 and NCBInr. The following search parameters were applied: trypsin, one missed cleavage, monoisotopic mass, carbamidomethyl (C) and oxidation (M) as variable modifications, ±0.3 Da precursor tolerance, ±0.3 Da MS/MS tolerance, 1 ^{13}C, +2/+3 peptide charge, ESI-TRAP as instrument type. For protein hits matched to uncultured species without taxonomic classification a protein–protein BLAST (blastp) search against NCBInr (version 2012/07/20) was carried out[21].

CARD analysis of *Microbacterium* sp. strain BR1's genome

The Comprehensive Antibiotic Resistance Database (CARD) analysis[22] of the 10 genome contigs of *Microbacterium* sp. strain BR1 was carried out on February 14th, 2017. The genome was screened with the Resistance Gene Identifier (RGI) for perfect and strict matches to known resistance genes.

Sequential protein purification

Microbacterium sp. strain BR1 cell suspensions with an OD_{600} of 10 were homogenized with a high pressure homogenizator (EmulsiFlex-B15, AVESTIN Europe GmbH, Mannheim, Germany). The homogenizator, buffer and the cell extract were precooled with ice-cold solutions and on ice, respectively. The pressure was set to 5.5 bar and the cell suspensions were homogenized four times with approximately 1 min breaks to chill the lysate on ice. Cell debris was removed from the extract by centrifugation at 60,000 × g and 4°C for 20 min.

The supernatant was cooled on ice and fractionated by the addition of ice-cold 100 % saturated ammonium sulphate stock solution to a final ammonium sulphate concentration of 40 %. The mixture was placed on ice to allow for precipitation under gentle stirring for 20 min. The sample was then centrifuged at 40,000 × g and 4 °C for 15 min and the supernatant was transferred to a new vial. The supernatant was brought to 70 % ammonium sulphate saturation by the addition of solid ammonium sulphate. After centrifugation (40'000 × g and 4 °C for 15 min), the pellet was resuspended in 5 ml of 20 mM Bis-Tris pH 7 and stored at -20 °C over night.

All following purification steps were performed at 4°C on a Pharmacia FPLC liquid chromatography system. For each purification step, fractions of 1 ml were collected and screened for activity with the NADH assay described below.

Hydrophobic interaction chromatography (HIC): A 1 ml HiTrap Phenyl HP column (GE Healthcare) was loaded with the resuspended 40-70% fraction of the ammonium sulphate precipitation. Fractions were then eluted using linear gradient from 20 % to 0 % saturated ammonium sulphate BisTris buffer (20 mM, pH 7) within 19 column volumes at 1 ml min^{-1}.

Weak anion exchange chromatography: Active fractions from the HIC purification were pooled and de-salted by ultrafiltration (Amicon Ultra, 10kDa molecular weight cut off, Sigma Aldrich). The retentate was diluted to a final volume of 2 ml in BisTris buffer (20 mM, pH 7) and loaded on a 1 ml HiTrap capto DEAE column (GE Healthcare). Proteins were eluted by a linear gradient from 0 to 1 M NaCl in BisTris buffer (20 mM, pH 7) in 10 column volumes at 1 ml min^{-1} was used for this separation step.

Strong anion exchange chromatography: Active fractions from the DEAE purification were pooled and desalted as described above. The sample was diluted to a final volume of 2 ml and loaded on a 1 ml Mono Q 5/50 GL column (GE Healthcare). Proteins were eluted by a linear gradient from 0 to 1 M NaCl in BisTris buffer (20 mM, pH 7) in 19 column volumes at 0.8 ml min^{-1}.

NADH assay used for activity screening of fractioned crude cell extract

Active fractions after FPLC were screened for their sulfonamide degrading activity by comparing NADH consumption rates of samples containing sulfadiazine (SDZ) to those without SDZ. NADH consumption rates were determined photometrically (Synergy 2, Biotek) in duplicates. Additionally, negative controls were tested in parallel, containing all ingredients of the assay except the fractionized sample (Table S 6). The NADH degradation rate was calculated based on the slope in the linear part of the kinetic (r^2 >= 0.98) assuming an ε_{NADH} at 340 nm of 6317 M^{-1} cm^{-1}. Sulfonamide specific NADH degradation rates were calculated subtracting the NADH consumption rates of controls without SDZ from the rates of samples containing SDZ.

Table S 6: Ingredients of the NADH assay for the screening of active cell extract fractions

Ingredients	Final concen-tration
SDZ	500 µM
NADH	1 mM
PBS pH 7.0	50 mM
NaCl	250 mM
Sample	30 % (v/v)
FMN*	2.5 µM
FRE (E.C.1.5.1.29 NovoCIB, Lyon, France)*	0.1 U ml^{-1}

*ingredients were added only if explicictly stated.

HPLC

A HPLC system series 1200 (Agilent Technologies, Germany) was used for all HPLC measurements. It was equipped with an auto-injector, a degasser, a diode array detector (DAD) and an on-line liquid scintillation radioflow detector (LSRD; Ramona Star; Raytest, Straubenhardt, Germany) with a cell volume of 1.3 ml. When required, the mass spectrometer (MS) MS 6320 Ion Trap HPLC/MS (Agilent) was used instead of the LSRD.

For the analysis of ^{14}C-labelled compounds, LSRD was coupled to the HPLC system in series with the DAD. This configuration allowed the assignment of a DAD signal to the corresponding ^{14}C signal. 50 µL of the samples were injected for DAD and/or LSRD detection. For LSRD, Ultima Flow^{TM} scintillation cocktail (Perkin Elmer, Waltham, USA) was used at a flow rate of 2.0 ml min^{-1}.

For HPLC/MS sample analysis the MS was connected in series to the DAD. For each HPLC/MS analysis, a sample volume of 5 µL was injected by the autosampler.

Analyses were carried out with methanol (eluent A) and H_2O with 0.1 % (v/v) formic acid (eluent B). Analytes were separated on a Nucleodur C18 pyramid 3 µm EC150/4 column from Macherey-Nagel (Düren, Germany) and a Macherey-Nagel CC 8/4 ND C18 Pyramid 3 µm guard column. The following gradient was used: 0 min 95 %B, 2 min 95% B, 8 min 41% B, 10 min 2% B, 12 min 95% B, with 3 min equilibration time before the next run. The flow rate was set to 0.8 ml min^{-1}.

SMX was either detected by DAD at 280 nm or by LSRD detector (for samples prepared with ^{14}C-SMX).

For the analysis of 4AP were performed with a gradient as follows: 0 to 1.5 min isocratic at 100 %B, then until 3 min decreasing to 85% B, 6 min 60% B, 8 min 0%, holding until 8.5, followed by 6 minutes of equilibration time at 100% B before the next run. The flow rate was set to 0.8 ml min^{-1} and detection at 270 nm.

GC-MS analysis of BQ, HQ and THB

Samples were analyzed on a 7890A series Agilent gas chromatograph (Basel, Switzerland) equipped with a Zebron ZB-5MS column, (30 m by 0.25 mm, 0.25 µ m film thickness, Phenomenex) coupled to an Agilent 5975C series mass spectrometer. The carrier gas was helium (1 ml min^{-1}). The injection volume was

1 µl (split 1:30). The temperature program was 70 °C for 3 min, 8 °C per minute to 250 °C; the injector temperature was 100 °C; the interface temperature 280 °C. The mass selective detector (EI) was operated in the scan mode (mass range *m/z* 50-600) with an electron energy of 70 eV.

Sample derivatization: This method is adapted from Kolvenbach *et al.*[23]. After the acidification of 300 µl aqueous sample with 30 µl 1 M HCl, analytes were extracted with 400 µl ethyl acetate. The organic phase was transferred into a fresh glass vial and dried with Na_2SO_4. 200 µl were transferred into a fresh GC glass vial and dried under a gentle nitrogen stream at 40 °C to complete dryness. Samples were resuspended in 100 µl ACN/BSTFA TCMS (80/20 v/v), before GC-MS analysis.

References:

1. Marengo, J. R., Kok, R. A., O'Brien, K., Velagaleti, R. R. & Stamm, J. M. Aerobic biodegradation of (14 C)-sarafloxacin hydrochloride in soil. *Environ. Toxicol. Chem.* **16,** 462–471 (1997).
2. Junker, T., Alexy, R., Knacker, T. & Kümmerer, K. Biodegradability of 14 C-Labeled Antibiotics in a Modified Laboratory Scale Sewage Treatment Plant at Environmentally Relevant Concentrations. *Environ. Sci. Technol.* **40,** 318–324 (2006).
3. Wehrhan, A. *Fate of veterinary pharmaceuticals in soil: An experimental and numerical study on the mobility, sorption and transformation of sulfadiazine.* (2006).
4. Schmidt, B. *et al.* Fate in soil of ^{14} C-sulfadiazine residues contained in the manure of young pigs treated with a veterinary antibiotic. *J. Environ. Sci. Heal. Part B* **43,** 8–20 (2008).
5. Henderson, K. L. D. Impact of veterinary antibiotics in the environment. (Iowa State University, 2008).
6. Bouju, H., Ricken, B., Beffa, T., Corvini, P. F.-X. & Kolvenbach, B. A. Isolation of bacterial strains capable of sulfamethoxazole mineralization from an acclimated membrane bioreactor. Appl. Environ. Microbiol. 78, 277–9 (2012).
7. Islas-Espinoza, M., Reid, B. J., Wexler, M. & Bond, P. L. Soil bacterial consortia and previous exposure enhance the biodegradation of sulfonamides from pig manure. Microb. Ecol. 64, 140–151 (2012).

8. Junge, T., Claßen, N., Schäffer, A. & Schmidt, B. Fate of the veterinary antibiotic 14C-difloxacin in soil including simultaneous amendment of pig manure with the focus on non-extractable residues. *J. Environ. Sci. Heal. Part B* **47,** 858–868 (2012).

9. Topp, E. et al. Accelerated biodegradation of veterinary antibiotics in agricultural soil following long-term exposure, and isolation of a sulfamethazine-degrading *Microbacterium* sp. J. Environ. Qual. 42, 173–178 (2013).

10. Tappe, W. et al. Degradation of sulfadiazine by *Microbacterium lacus* strain SDZm4 isolated from lysimeters previously manured with slurry from sulfadiazine medicated pigs. Appl. Environ. Microbiol. 79, 2572–7 (2013).

11. Jessick, A. M., Moorman, T. B. & Coats, J. R. Fate of Erythromycin in Sediment-Containing Surface Water Microcosms: How Does Aged Erythromycin in Sediment Influence Bioavailability? *Entomol. Publ. - Iowa State Univ.* 161–178 (2013). doi:10.1021/bk-2013-1126.ch007

12. Reis, P. J. M. et al. Biodegradation of sulfamethoxazole and other sulfonamides by Achromobacter denitrificans PR1. J. Hazard. Mater. 280, 741–9 (2014).

13. Kim, Y. H., Pak, K., Pothuluri, J. V. & Cerniglia, C. E. Mineralization of erythromycin A in aquaculture sediments. *FEMS Microbiol. Lett.* **234,** 169–175 (2004).

14. Topp, E., Renaud, J., Sumarah, M. & Sabourin, L. Reduced persistence of the macrolide antibiotics erythromycin, clarithromycin and azithromycin in agricultural soil following several years of exposure in the field. *Sci. Total Environ.* **562,** 136–144 (2016).

15. Veltri, D., Wight, M. M. & Crouch, J. A. SimpleSynteny: a web-based tool for visualization of microsynteny across multiple species. *Nucleic Acids Res.* **44,** W41–W45 (2016).

16. Heyer, R. *et al.* Metaproteome analysis of the microbial communities in agricultural biogas plants. *N. Biotechnol.* **30,** 614–22 (2013).

17. Schweikl, H., Klein, U., Schindlbeck, M. & Wieczorek, H. A vacuolar-type ATPase, partially purified from potassium transporting plasma membranes of tobacco hornworm midgut. *J. Biol. Chem.* **264,** 11136–11142 (1989).

18. Laemmli, U. K. Cleavage of Structural Proteins during the Assembly of the Head of Bacteriophage

T4. *Nature* **227,** 680–685 (1970).

19. Shevchenko, A., Wilm, M., Vorm, O. & Mann, M. Mass Spectrometric Sequencing of Proteins from Silver-Stained Polyacrylamide Gels. *Anal. Chem.* **68,** 850–858 (1996).

20. Perkins, D. N., Pappin, D. J. C., Creasy, D. M. & Cottrell, J. S. Probability-based protein identification by searching sequence databases using mass spectrometry data. *Electrophoresis* **20,** 3551–3567 (1999).

21. Altschul, S. F. et al. Gapped BLAST and PSI-BLAST: a new generation of protein database search programs. Nucleic Acids Res. 25, 3389–3402 (1997).

22. Jia, B. et al. CARD 2017: expansion and model-centric curation of the comprehensive antibiotic resistance database. Nucleic Acids Res. 45, D566–D573 (2017).

23. Kolvenbach, B. A. *et al.* Purification and characterization of hydroquinone dioxygenase from Sphingomonas sp. strain TTNP3. *AMB Express* **1,** 8 (2011).
